# Endothelial MICU1 protects against vascular inflammation and atherosclerosis by inhibiting mitochondrial calcium uptake

**DOI:** 10.1172/JCI181928

**Published:** 2025-04-01

**Authors:** Lu Sun, Ruixue Leng, Monan Liu, Meiming Su, Qingze He, Zhidan Zhang, Zhenghong Liu, Zhihua Wang, Hui Jiang, Li Wang, Shuai Guo, Yiming Xu, Yuqing Huo, Clint L. Miller, Maciej Banach, Yu Huang, Paul C. Evans, Jaroslav Pelisek, Giovanni G. Camici, Bradford C. Berk, Stefan Offermanns, Junbo Ge, Suowen Xu, Jianping Weng

**Affiliations:** 1 Department of Endocrinology, The First Affiliated Hospital of USTC, Division of Life Sciences and Medicine, University of Science and Technology of China, Hefei, Anhui, China.; 2 Department of Pharmacy, The First Affiliated Hospital of USTC, Division of Life Sciences and Medicine, University of Science and Technology of China, Hefei, Anhui, China.; 3 Department of Epidemiology and Biostatistics, School of Public Health, Anhui Medical University, Hefei, Anhui, China.; 4 Department of Biomedical Sciences, City University of Hong Kong, Hong Kong, China.; 5 School of Basic Medical Sciences, State Key Lab of Respiratory Disease, Guangzhou Medical University, Guangzhou, Guangdong, China.; 6 Department of Molecular and Cellular Biology, Baylor College of Medicine, Houston, Texas, USA.; 7 Center for Public Health Genomics, University of Virginia, Charlottesville, Virginia, USA.; 8 Department of Preventive Cardiology and Lipidology, Medical University of Lodz (MUL), Lodz, Poland.; 9 Centre for Biochemical Pharmacology, William Harvey Research Institute, Barts and The London Faculty of Medicine and Dentistry, Queen Mary University of London, London, United Kingdom.; 10 Department of Vascular Surgery, University Hospital Zurich, Zurich, Switzerland.; 11 Center for Molecular Cardiology, University of Zurich, Schlieren, Switzerland.; 12 Aab Cardiovascular Research Institute, Department of Medicine, University of Rochester School of Medicine and Dentistry, Rochester, New York, USA.; 13 Department of Pharmacology, Max Planck Institute for Heart and Lung Research, Bad Nauheim, Germany.; 14 Department of Cardiology, Zhongshan Hospital, Fudan University, Shanghai Institute of Cardiovascular Diseases, Shanghai, China.; 15 Anhui Provincial Key Laboratory of Metabolic Health and Panvascular Diseases, Hefei, Anhui, China.; 16 Department of Endocrinology, The First Affiliated Hospital of Anhui Medical University, Hefei, Anhui, China.

**Keywords:** Cell biology, Vascular biology, Atherosclerosis

## Abstract

Mitochondrial dysfunction fuels vascular inflammation and atherosclerosis. Mitochondrial calcium uptake 1 (MICU1) maintains mitochondrial Ca^2+^ homeostasis. However, the role of MICU1 in vascular inflammation and atherosclerosis remains unknown. Here, we report that endothelial MICU1 prevents vascular inflammation and atherosclerosis by maintaining mitochondrial homeostasis. We observed that vascular inflammation was aggravated in endothelial cell–specific *Micu1* knockout mice (*Micu1*^ECKO^) and attenuated in endothelial cell–specific *Micu1* transgenic mice (*Micu1*^ECTg^). Furthermore, hypercholesterolemic *Micu1*^ECKO^ mice also showed accelerated development of atherosclerosis, while *Micu1*^ECTg^ mice were protected against atherosclerosis. Mechanistically, MICU1 depletion increased mitochondrial Ca^2+^ influx, thereby decreasing the expression of the mitochondrial deacetylase sirtuin 3 (SIRT3) and the ensuing deacetylation of superoxide dismutase 2 (SOD2), leading to the burst of mitochondrial reactive oxygen species (mROS). Of clinical relevance, we observed decreased MICU1 expression in the endothelial layer covering human atherosclerotic plaques and in human aortic endothelial cells exposed to serum from patients with coronary artery diseases (CAD). Two-sample Wald ratio Mendelian randomization further revealed that increased expression of MICU1 was associated with decreased risk of CAD and coronary artery bypass grafting (CABG). Our findings support MICU1 as an endogenous endothelial resilience factor that protects against vascular inflammation and atherosclerosis by maintaining mitochondrial Ca^2+^ homeostasis.

## Abstract 2

The present study depicts the role of MICU1 in atheroprotection by maintaining mitochondrial homeostasis and preventing vascular inflammation.

## Graphical abstract

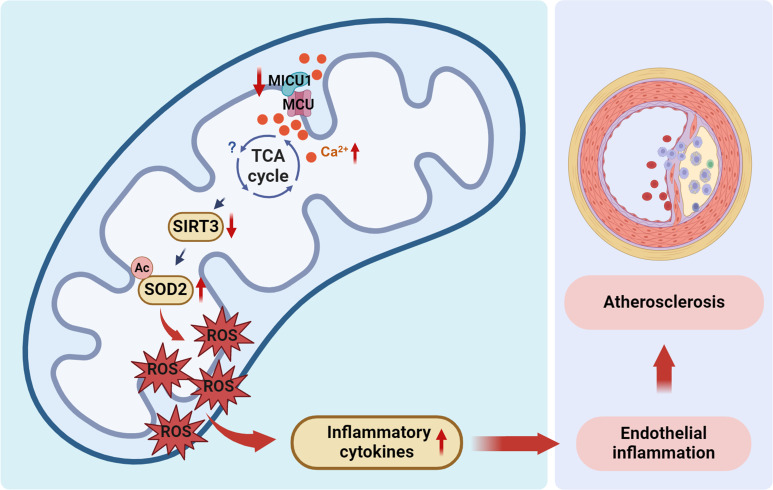

## Introduction

Atherosclerotic cardiovascular disease (ASCVD) remains the leading cause of death worldwide (1). Vascular inflammation is an early independent risk factor for ASCVD, and targeting inflammation can reduce cardiovascular events in high-risk patients (2–4). Endothelial cells (ECs) are a key regulator of vascular inflammation. Upon activation by inflammatory mediators, ECs respond through an increased expression of leukocyte adhesion molecules and proinflammatory cytokines, which consequently recruit inflammatory cells from the circulation to the artery wall (5). Thus, the protection of the endothelium from inflammation may be a viable strategy in preventing vascular inflammation and atherosclerosis.

Vascular inflammation can result from mitochondrial instability (6). Normally, proper function of mitochondria regulates cellular metabolism and energy generation in mammalian physiology by being the principal source of ATP. However, under pathological conditions, dysfunctional mitochondria lead to Ca^2+^ overload and reactive oxygen species (ROS) production, which acts upstream of multiple kinases and signaling cascades. The net outcome is energy metabolic disorder, cell damage (7, 8), and inflammation (9, 10). Thus, fine-tuning of Ca^2+^ levels is essential for mitochondrial homeostasis and antiinflammation (8). Ca^2+^ uptake into mitochondria is tightly regulated by mitochondrial calcium uptake 1 (MICU1), the first reported member of the mitochondrial calcium uniporter complex (mtCU) (11). The mtCU consists of a Ca^2+^ uptake channel called mitochondrial calcium uniporter (MCU) (12, 13), MICU1-3 as regulatory subunits (11, 14), MCUb, and essential MCU regulator (EMRE) (15, 16). MICU1 serves as an essential gatekeeper of MCU in a closed conformation at low cytosolic Ca^2+^ concentrations to regulate mitochondria signaling and function (11, 17–20). MICU1 has known roles in liver regeneration, lung alveolar type 2 cell plasticity, and neurodegeneration (21–23), but its role in vascular pathophysiology remains elusive.

In this study, we investigated the potential role of endothelial MICU1 in vascular inflammation and atherosclerosis by focusing on mitochondrial function, Ca^2+^ dynamics, and redox balance. We observed that MICU1 depletion augmented inflammatory responses in human venous and arterial ECs. In addition, EC-specific *Micu1*-knockout mice (*Micu1*^ECKO^) accelerated, while EC-specific *Micu1* transgenic mice (*Micu1*^ECTg^) were protected against ROS generation, vascular inflammation, and atherosclerosis. Mechanistically, MICU1 restrains endothelial inflammation via the sirtuin 3 (SIRT3)/superoxide dismutase 2 (SOD2)/mitochondrial reactive oxygen species (mROS) pathway. Of clinical significance, MICU1 expression was decreased in the human plaque region and increased expression of MICU1 was associated with decreased risk of coronary artery disease (CAD) and coronary artery bypass grafting (CABG). Thus, MICU1 represents an endogenous resilience factor against vascular inflammation and atherosclerosis in the context of mitochondrial dysfunction.

## Results

### RNA-Seq profiling reveals the antiinflammatory role of MICU1 in ECs.

**Figure 1:**
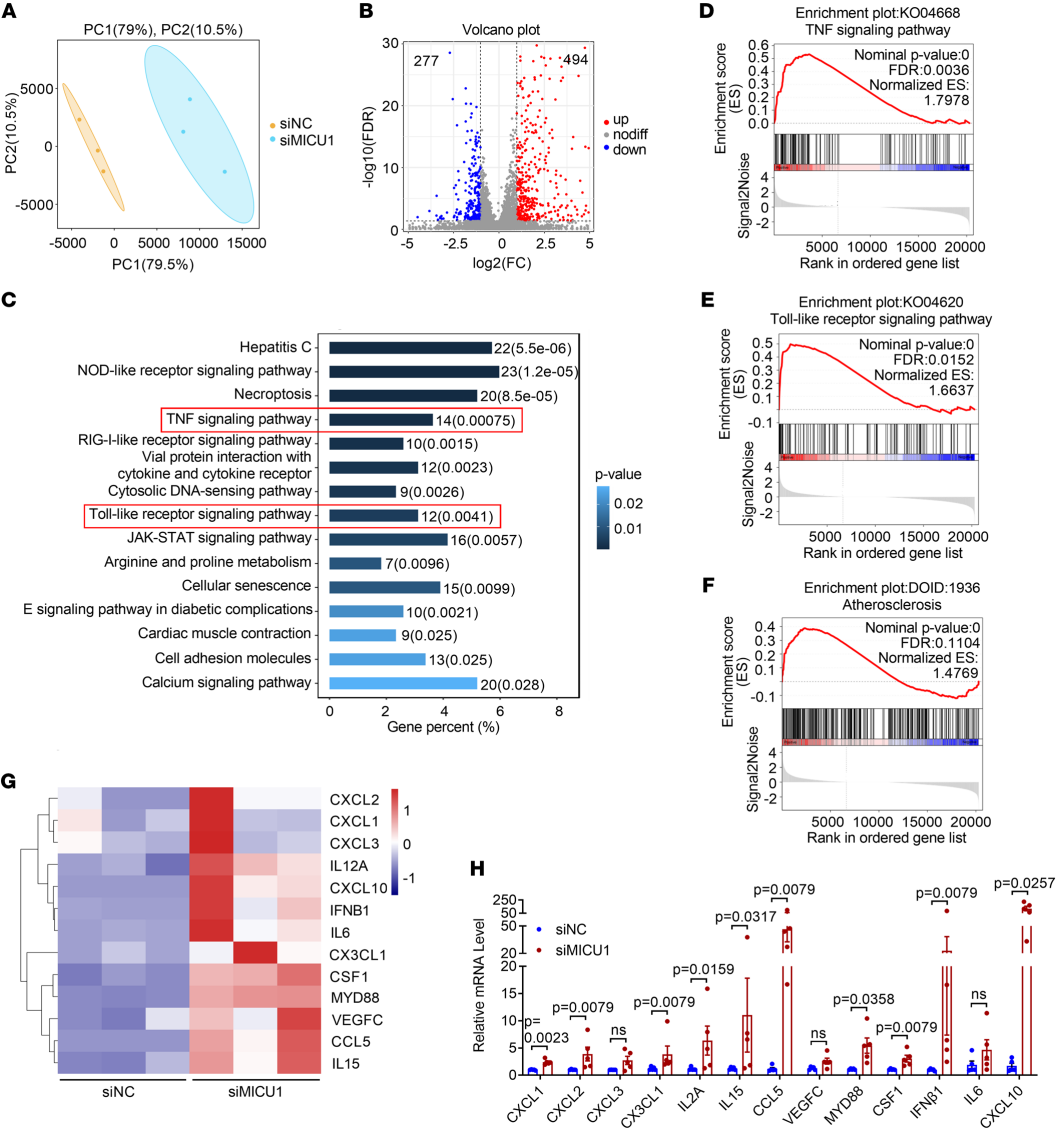
RNA-Seq profiling reveals the anti-inflammatory role of MICU1 in ECs. (**A**) Principal component analysis (PCA) comparing the transcriptomic data and plotting by coordinates for principal component 1 (PC1) and PC2. Color coding was used to separate treatment with negative control siRNA (siNC) or MICU1 siRNA (siMICU1). (**B**) Volcano plot showing differentially expressed genes after knockdown of MICU1 in HUVECs. Selection criteria: gene expression fold change (FC) ≥ 2 and FDR < 0.05. (**C**) KEGG enrichment for differentially expressed genes after MICU1 depletion. (**D**–**F**) GSEA analysis was used to examine the enrichment of the TNF-signaling pathway (**D**),the TLR-signaling pathway (**E**), and atherosclerosis (**F**). (**G**) Heatmap showing the key differentially expressed genes from the KEGG top pathway related to inflammatory chemokines or cytokines. (**H**) qRT-PCR analysis of mRNA levels of inflammatory chemokines and cytokines after MICU1 depletion (*n* = 5). Statistical analysis was performed by Welch’s *t* test (CXCL1, CXCL3, VEGFC, MYD88, and CXCL10 of **H**) and Mann-Whitney *U* test (CXCL2, CX3CL1, IL2A, IL15, CCL5, CSF1, IFNβ1, and IL-6 of **H**).

To understand the potential role of *MICU1* in endothelial function, we performed a transcriptomic profiling study in HUVECs (Figure 1A). Silencing of *MICU1* significantly increased the expression of 494 genes while decreasing that of 277 genes (based on fold change ≥ 2 and FDR < 0.05; Figure 1B). Differentially expressed genes were associated with multiple Kyoto Encyclopedia of Genes and Genomes (KEGG) pathways linked to inflammatory responses, including TNF signaling, TLR signaling, NOD-like receptor signaling, RIG-I–like receptor signaling, and JAK-STAT signaling (Figure 1C). Similarly, gene set enrichment analysis (GSEA) showed that MICU1 depletion upregulated TNF signaling, TLR signaling (Figure 1, D and E), and atherosclerosis-related pathways (Figure 1F). Consistently, the expression of multiple inflammatory cytokines and chemokines including *CXCL1*, *CXCL2*, *CXCL3*, C-X3-C motif chemokine ligand 1 (*CX3CL1*), *IL12A*, *IL15*, *CCL5*, *VEGFC*, *MYD88*, *CSF1*, *IFN*β*1*, *IL6*, and *CXCL10* were increased in HUVECs after *MICU1* depletion compared with negative control (Figure 1G). These data were validated by real-time quantitative reverse transcription PCR (qRT-PCR) which confirmed that MICU1 depletion increased the expression of multiple chemokines and cytokines (Figure 1H). These results indicate that MICU1 serves as an endogenous protective factor in controlling basal expression of inflammatory molecules.

### MICU1 reduces EC inflammation in vitro.

To further address the role of MICU1 in inflammatory response, we first performed an in vivo experiment to explore the influence of inflammatory treatment (LPS) on MICU1 expression in endothelium. En face immunofluorescence staining showed that MICU1 expression in aortic endothelium was reduced in mice treated with LPS for 6 hours compared with control (Supplemental Figure 1; supplemental material available online with this article; https://doi.org/10.1172/JCI181928DS1). We hypothesized that inflammatory stimulation with LPS reduces the expression of MICU1, which may further propagate the inflammatory activation cascade.

**Figure 2:**
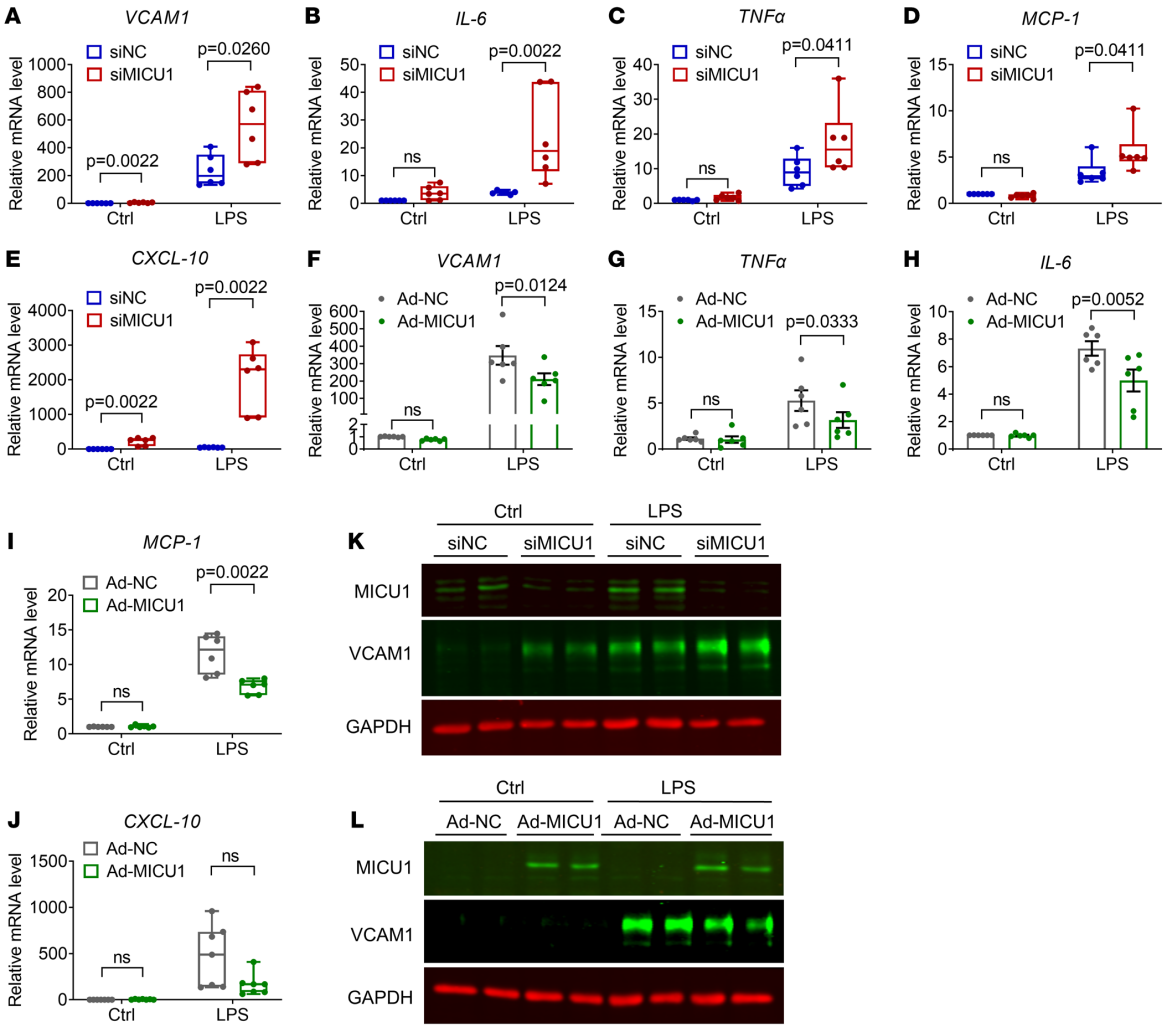
MICU1 attenuates EC inflammation in vitro. (**A**–**E**) qRT-PCR analysis of mRNA levels of VCAM1 (**A**, *n* = 6), IL-6 (**B**, *n* = 6), TNF-α (**C**, *n* = 6), MCP-1 (**D**, *n* = 6), and CXCL-10 (**E**, *n* = 6) in HUVECs after treatment with siNC or siMICU1 in the presence of LPS (1 μg/ml) for 6 hours. (**F**–**J**) HUVECs were transfected with negative control adenovirus (Ad-NC) or MICU1 adenovirus (Ad-MICU1) before qRT-PCR analysis of mRNA levels of VCAM1 (**F**, *n* = 6), TNF-α (**G**, *n* = 6), IL-6 (**H**, *n* = 6), MCP-1 (**I**, *n* = 6), and CXCL-10 (**J**, *n* = 7). Cells were exposed to LPS (1 μg/ml) for 6 hours. (**K**) The expression of VCAM1 was detected by immunoblot in HAECs. Cells were treated with siNC or siMICU1 and then exposed to LPS (1 μg/ml) for 6 hours (*n* = 4). Data shown are from 2 different donors. (**L**) The expression of VCAM1 was detected by immunoblot in HAECs. Cells were treated with Ad-NC or Ad-MICU1 and then exposed to LPS (1 μg/ml) for 6 hours (*n* = 4). Data shown are from 2 different donors. Statistical analysis was performed by multiple Mann-Whitney *U* tests (**A**–**E**, **I**, and **J**) and 2-way ANOVA followed by Bonferroni’s post hoc tests (**F**–**H**).

To validate this hypothesis, we next assessed the effect of gain and loss of function of *MICU1* on the expression of inflammatory genes in ECs treated with LPS. We observed that silencing of *MICU1* significantly elevated the expression of *VCAM1*, *IL-6*, *TNF*α, monocyte chemoattractant protein 1 (*MCP-1*), and *CXCL-10* in LPS-treated HUVECs (Figure 2, A–E), whereas, overexpression of MICU1 produced the opposite effects except that *CXCL-10* expression was not changed (Figure 2, F–J). Also, siRNA-mediated MICU1 depletion increased, while adenovirus-mediated MICU1 overexpression decreased VCAM1 protein expression (Figure 2, K and L). Collectively, these results support that MICU1 negatively regulates EC inflammation.

### MICU1 reduces EC inflammation in vivo.

Prompted by the antiinflammatory role of MICU1 in vitro, we sought to explore its role in vivo. We first generated EC-specific *Micu1*-knockout mice (*Micu1*^ECKO^) by crossbreeding *Micu1*^fl/fl^ mice with *Cdh5-Cre* mice (Supplemental Figure 2A). Successful deletion of *Micu1* in *Micu1*^ECKO^ mice is shown in Supplemental Figure 2, B and C. En face immunofluorescence staining also confirmed the ablation of endothelial MICU1 in *Micu1*^ECKO^ mice (Supplemental Figure 2D). *Micu1*^ECKO^ mice have no appreciable physiological defects compared with *Micu1*^fl/fl^ littermate controls. The baseline characterizations were similar between *Micu1*^ECKO^ mice and *Micu1*^fl/fl^ mice (Supplemental Figure 3).

**Figure 3:**
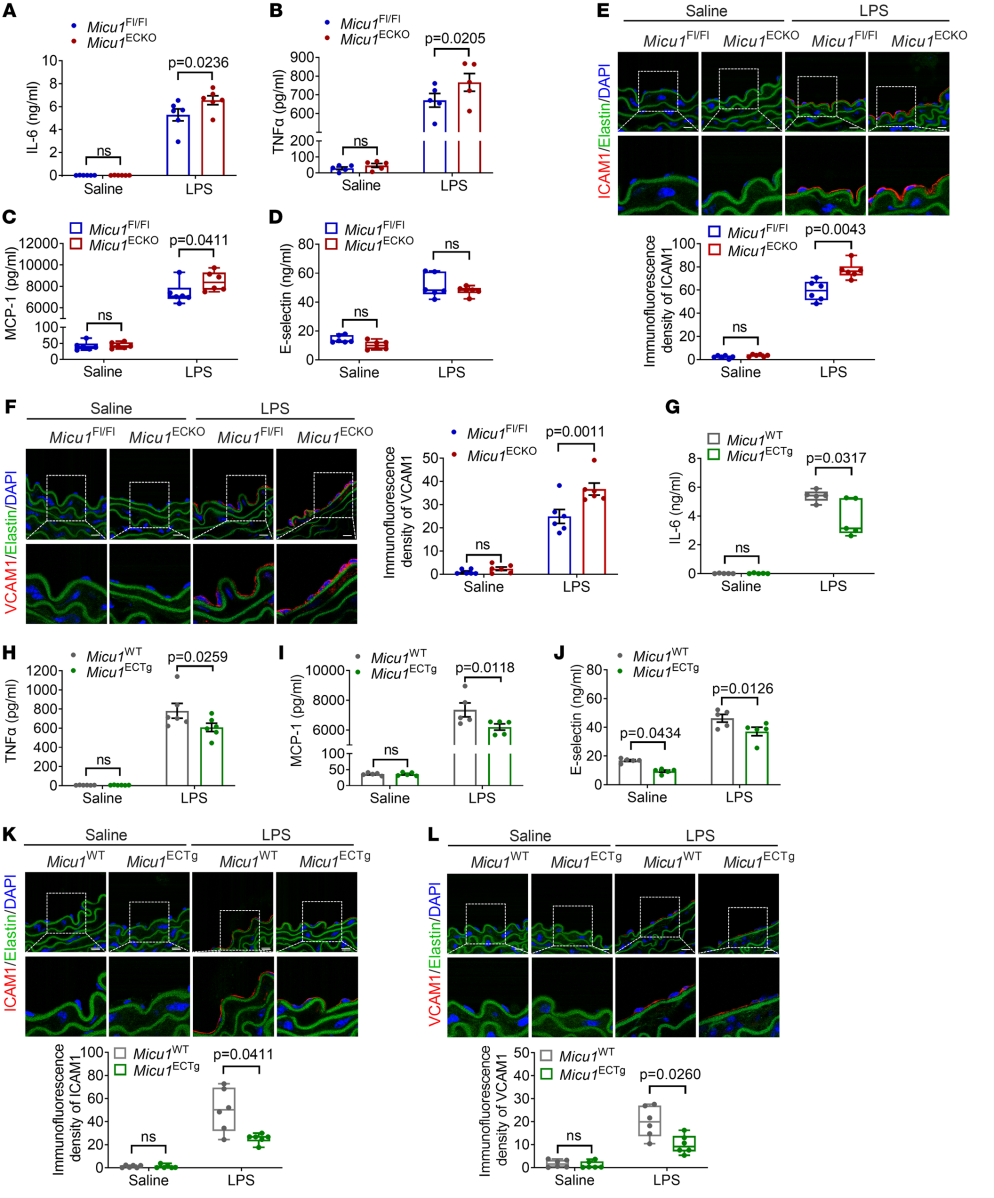
MICU1 attenuates EC inflammation in vivo. (**A**–**D**) ELISA of IL-6 (**A**, *n* = 6), TNF-α (**B**, *n* = 5), MCP-1 (**C**, *n* = 6), and E-selectin (**D**, *n* = 6) in serum from *Micu1*^fl/fl^ mice or *Micu1*^ECKO^ mice treated with saline or LPS (10 mg/kg) for 6 hours. (**E** and **F**) Representative confocal microscopy images of ICAM1 expression (**E**, *n* = 6) and VCAM1 expression (**F**, *n* = 6) in the aortic sections of *Micu1*^fl/fl^ mice or *Micu1*^ECKO^ mice exposed to saline or LPS (10 mg/kg) for 6 hours. ICAM1 or VCAM1 (red), elastin (green), and DAPI (blue). Scale bars: 10 μm. (**G**–**J**) ELISA of IL-6 (**G**, *n* = 5), TNF-α (**H**, *n* = 6), MCP-1 (**I**, *n* = 5), and **E**-selectin (**J**, *n* = 5) in serum from *Micu1*^WT^ mice or *Micu1*^ECTg^ mice exposed to saline or LPS (10 mg/kg) for 6 hours. (**K** and **L**) Representative images of ICAM1 (**K**, *n* = 6) and VCAM1 protein expression (**L**, *n* = 6) in the aortic sections of *Micu1*^WT^ mice or *Micu1*^ECTg^ mice exposed to saline or LPS (10 mg/kg) for 6 hours. Scale bars: 10 μm. Original magnification, ×80. Statistical analysis was performed by 2-way ANOVA followed by Bonferroni’s post hoc tests (**A**, **B**, **F**, and **H**–**J**) and multiple Mann-Whitney *U* tests (**C**–**E**, **G**, **K**, **L**).

Next, the mice were injected with LPS for 6 hours to induce vascular inflammation. *Micu1*^ECKO^ mice exhibited significantly elevated levels of IL-6, TNF-α, and MCP-1 after LPS treatment (Figure 3, A–C), whereas the expression of E-selectin remained unaffected (Figure 3D). Furthermore, confocal microscopy showed increased ICAM1 and VCAM1 protein expression in *Micu1*^ECKO^ mouse aortic endothelium compared with control *Micu1*^fl/fl^ mice after LPS treatment (Figure 3, E and F). These findings demonstrate that *Micu1* deficiency in vascular endothelium aggravated the LPS-induced inflammatory response.

To determine whether *Micu1* gain of function prevents LPS-induced vascular inflammation, we generated EC-specific *Micu1* transgenic mice (*Micu1*^ECTg^) by crossbreeding *Rosa26*^LSL-Micu1^ mice with *Cdh5*-Cre mice (Supplemental Figure 4A). Mice were validated by tail genotyping and qRT-PCR for the mRNA level of *Micu1* in the aortic endothelium (Supplemental Figure 4, B and C). En face immunofluorescence staining showed successful overexpression of MICU1 in aortic endothelium (Supplemental Figure 4D). Phenotypically, *Micu1*^ECTg^ mice behaved normally and were viable with baseline phenotypes comparable to those of littermate controls (Supplemental Figure 5). Interestingly, LPS-induced increase of IL-6, TNF-α, MCP-1, and E-selectin was significantly attenuated in *Micu1*^ECTg^ mice compared with control mice (Figure 3, G–J). Moreover, immunofluorescent staining revealed decreased ICAM1 and VCAM1 protein expression in the aortic endothelium of *Micu1*^ECTg^ mice compared with control mice exposed to LPS (Figure 3, K and L). These results demonstrate that *Micu1* overexpression protects vascular endothelium from LPS-induced inflammatory response in a mouse model. Overall, these sets of data demonstrate that MICU1 is a negative regulator of endothelial activation and vascular inflammation in vivo.

### MICU1 regulates mitochondrial Ca^2+^ uptake and ROS production in ECs.

**Figure 4:**
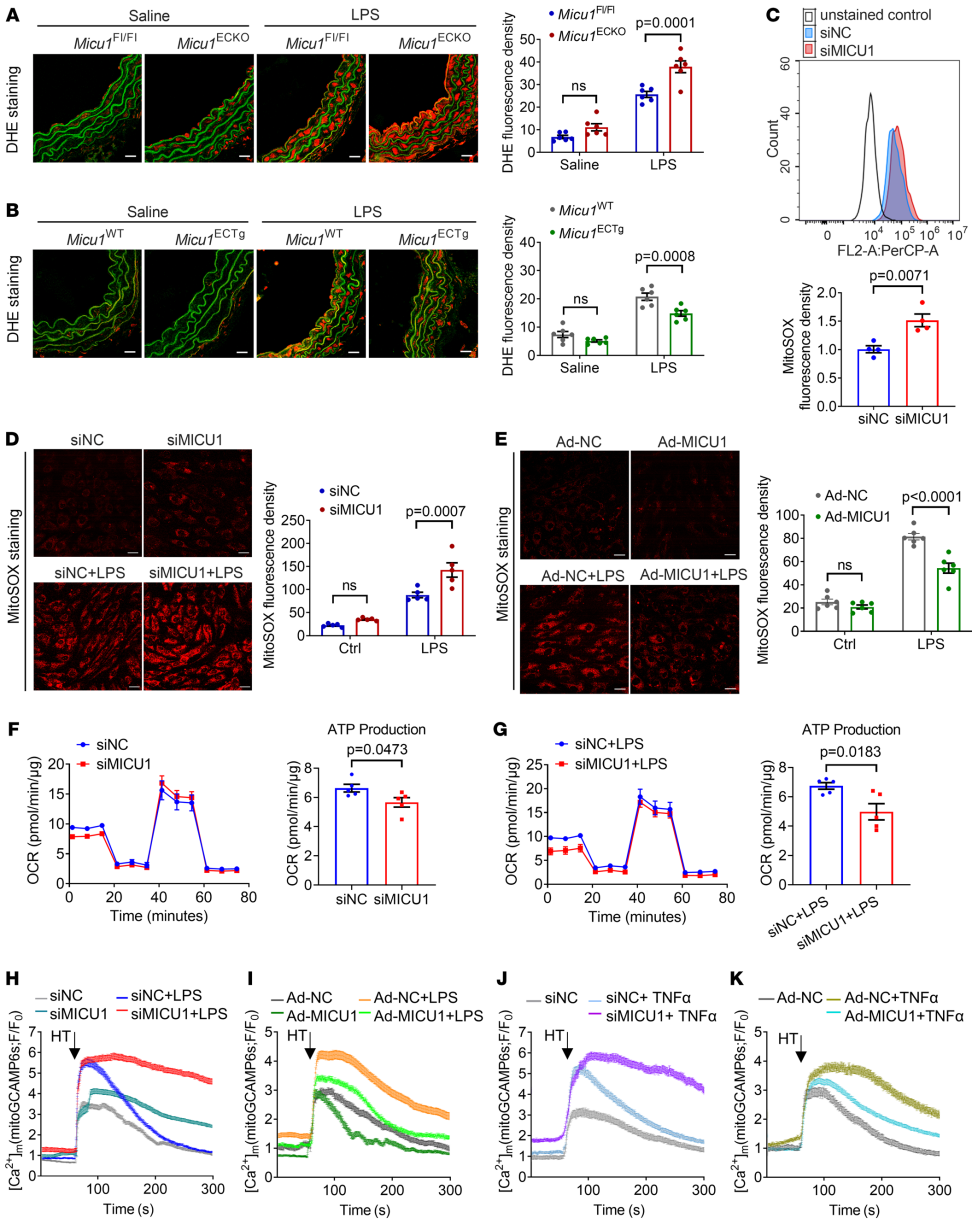
MICU1 regulates mitochondrial Ca^2+^ uptake and ROS production in ECs. (**A** and **B**) Dihydroethidium (DHE) staining of aortic sections from *Micu1*^fl/fl^ mice or *Micu1*^ECKO^ mice (**A**), *Micu1*^WT^ mice or *Micu1*^ECTg^ mice (**B**) treated with saline or LPS (10 mg/kg) for 6 hours (*n* = 6). Scale bars: 20 μm. (**C**) Flow cytometry analysis of mROS levels using MitoSOX (*n* = 4). (**D** and **E**) MitoSOX fluorescence in HAECs depleted of MICU1 (**D**, *n* = 5) or overexpressed MICU1 (**E**, *n* = 6). Scale bars: 20 μm. (**F**) OCR of HAECs transfected with siNC or siMICU1. ATP production-linked OCR was analyzed and quantified. (**G**) OCR of HAECs transfected with siNC or siMICU1 in the presence of LPS (1μg/ml, 16 hours). ATP production-linked OCR was analyzed and quantified. (**H**) Kinetics of [Ca^2+^]_m_ in response to HT (50 μM) in HAECs treated with siNC or siMICU1 in the presence (siNC+LPS, *n* = 30 cells; siMICU1+LPS, *n* = 10 cells) or absence (siNC, *n* = 15 cells; siMICU1, *n* = 10 cells) of LPS (1μg/ml) for 6 hours. (**I**) Kinetics of [Ca^2+^]_m_ in response to HT (50 μM) in HAECs treated with Ad-NC or Ad-MICU1 in the presence (Ad-NC+LPS, *n* = 11 cells; Ad-MICU1+LPS, *n* = 15 cells) or absence (Ad-NC, *n* = 8 cells; Ad-MICU1, *n* = 13 cells) of LPS (1μg/ml) for 6 hours. (**J** and **K**) Kinetics of [Ca^2+^]_m_ in response to HT (50 μM) in HAECs treated with siNC or siMICU1 (**J**), Ad-NC, or Ad-MICU1 (**K**) in the presence or absence of TNF-α (10 ng/ml) for 6 hours (siNC, *n* = 10 cells; siNC+TNF-α, *n* = 14 cells; siMICU1+TNF-α, *n* = 20 cells; Ad-NC, *n* = 13 cells; Ad-NC +TNF-α, *n* = 16 cells; Ad-MICU1+TNF-α, *n* = 27 cells). Statistical analysis was performed by 2-way ANOVA followed by Bonferroni’s post hoc tests (**A**, **B**, **D**, and **E**) and Student’s *t* test (**C**, **F**, and **G**).

It is well established that ROS overproduction drives vascular inflammation. To understand the mechanisms whereby MICU1 regulates endothelial inflammation, we examined ROS levels in vivo and in vitro. EC-specific *Micu1* deletion led to excessive ROS levels of aortic sections in response to LPS treatment in mice (Figure 4A), while, aortic sections from EC-specific *Micu1* transgenic mice showed decreased ROS levels (Figure 4B). Then, we performed flow cytometry to quantify the role of MICU1 in mROS generation using mitochondrial superoxide indicator (mitoSOX) staining. Our data indicated that MICU1 silencing led to increased mROS generation (Figure 4C). Consistently, the production of mROS was enhanced by MICU1 depletion and was abrogated by MICU1 overexpression both in human aortic ECs (HAECs) (Figure 4, D and E) and HUVECs (Supplemental Figure 6, A and B). Additionally, intracellular ROS levels in HUVECs also showed a pattern of change similar to that of mROS (Supplemental Figure 6, C and D).

We then explored whether MICU1 regulates mitochondrial bioenergetics via controlling Ca^2+^ uptake (7, 19, 24). The oxygen consumption rate (OCR) was measured in real time to evaluate mitochondrial respiration in ECs. We found that, whether in the presence or absence of inflammatory stimuli, silencing MICU1 did not significantly affect the maximal OCR (Supplemental Figure 7, A and B). However, it markedly reduced the ATP production-linked OCR under basal and LPS-stimulated conditions, underscoring the necessity of MICU1 for mitochondrial ATP production (Figure 4, F and G). We then assessed the effects of MICU1 on mitochondrial Ca^2+^ uptake. Strikingly, [Ca^2+^]_m_ in response to the agonist histamine (HT) was enhanced by MICU1 silencing under both basal conditions and LPS treatment (Figure 4H). In contrast, overexpression of MICU1 attenuated mitochondrial Ca^2+^ uptake in response to HT compared with control (Figure 4I). Moreover, the effects of MICU1 silencing or overexpression under TNF-α treatment were consistent with the results of LPS treatment (Figure 4, J and K), underscoring the universal regulatory role of MICU1 in Ca^2+^ dynamics. These results indicate that [Ca^2+^]_m_ accumulation in ECs following inflammatory treatment could be reduced by MICU1, therefore contributing to ROS reduction and inhibition of vascular inflammation.

### MICU1 regulates EC inflammation via [Ca^2+^]_m_ and the SIRT3/SOD2 pathway.

**Figure 5:**
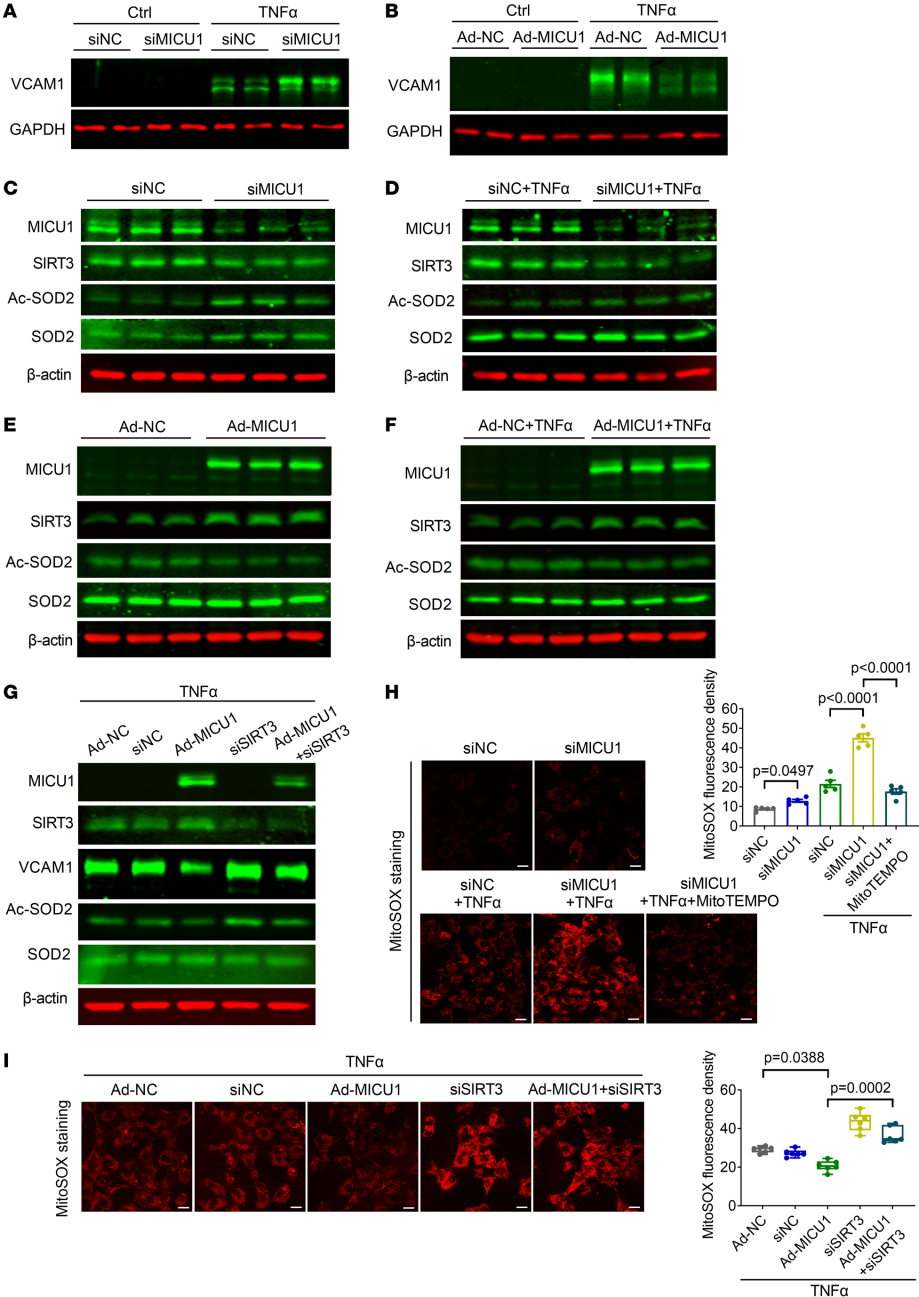
MICU1 regulates EC inflammation via [Ca^2+^]_m_ and SIRT3/SOD2 pathway. (**A**) Protein expression of VCAM1 was determined by immunoblot in HAECs. Cells were treated with siNC or siMICU1 and then exposed to TNF-α (10 ng/ml) for 6 hours (*n* = 6). (**B**) Protein expression of VCAM1 was determined by immunoblot in HAECs. Cells were treated with Ad-NC or Ad-MICU1 and then exposed to TNF-α (10 ng/ml) for 6 hours (*n* = 6). (**C** and **D**) Protein expression of SIRT3 and Ac-SOD2 were determined by immunoblot after MICU1 silencing in HAECs in the presence or absence of TNF-α (10 ng/ml) for 6 hours (*n* = 6). (**E** and **F**) Protein expression of SIRT3 and Ac-SOD2 were determined by immunoblot after MICU1 overexpression in HAECs in the presence or absence of TNF-α (10 ng/ml) for 6 hours (*n* = 6). (**G**) Protein expression of SIRT3, Ac-SOD2, and VCAM1 in HAECs was determined by immunoblot with SIRT3 silencing and MICU1 overexpression concurrently. Cells were exposed to TNF-α (10 ng/ml) for 6 hours (*n* = 5). (**H**) Representative images showing MitoSOX fluorescence in HAECs transfected with siNC or siMICU1 and then exposed to TNF-α (10 ng/ml). MitoTEMPO (5 μM) was added for 1 hour (*n* = 5). (**I**) Representative images showing MitoSOX fluorescence in HAECs with SIRT3 silencing or MICU1 overexpression or SIRT3 silencing and MICU1 overexpression concurrently and then exposed to TNF-α (10 ng/ml) (*n* = 6). Scale bars: 20 μm. Statistical analysis was performed by 1-way ANOVA followed by Bonferroni’s post hoc tests (**H**) and Kruskal-Wallis test followed by Dunn’s multiple-comparisons test (**I**).

To define the mechanisms linking MICU1-regulated [Ca^2+^]_m_ to ROS production, we investigated the potential role of SIRT3, given this enzyme can couple [Ca^2+^]_m_ with mROS by targeting SOD2 for deacetylation (25, 26). We next confirmed the influence of MICU1 on the inflammatory marker VCAM1 in HAECs. MICU1 silencing with TNF-α treatment led to elevated VCAM1 expression, while MICU1 overexpression decreased it (Figure 5, A and B). It is noteworthy that SIRT3 expression was decreased in response to MICU1 silencing in HAECs under basal and TNF-α–stimulated conditions (Figure 5, C and D), which could be reversed by MICU1 overexpression (Figure 5, E and F). Furthermore, MICU1 depletion led to an increase of acetylated SOD2, but did not alter total SOD2 levels (indicating SOD2 hyperacetylation) (Figure 5, C and D). In contrast, overexpression of MICU1 reduced SOD2 acetylation (Figure 5, E and F). To further examine the role of SIRT3 in endothelial dysfunction caused by *MICU1* depletion, we silenced *SIRT3* in HAECs. Western blot analysis showed that overexpression of MICU1 reduced VCAM1 protein expression which was reversed by SIRT3 depletion (Figure 5G). These results suggest that endothelial MICU1 regulates the SIRT3/SOD2 pathway.

Furthermore, MICU1 silencing caused elevated mROS levels, which could be scavenged by mitoTEMPO (a mitochondrial targeted antioxidant and a specific scavenger of mitochondrial superoxide) (Figure 5H). In addition, MICU1 overexpression led to decreased mROS levels, while SIRT3 knockdown blunted the effect of MICU1 overexpression on HAECs (Figure 5I). These data suggest a critical mediator role of SIRT3 in the protective effects executed by MICU1.

### MICU1 deletion in ECs aggravates atherosclerosis.

Since inflammation drives atherosclerosis (27, 28), we hypothesized that endothelial MICU1 may influence this process. To test this, *Micu1*^ECKO^ mice and *Micu1*^fl/fl^ mice were treated with AAV8-PCSK9^D377Y^ concurrent with a Western-type diet, feeding for 12 weeks to accelerate hypercholesterolemia and atherosclerosis.

**Figure 6:**
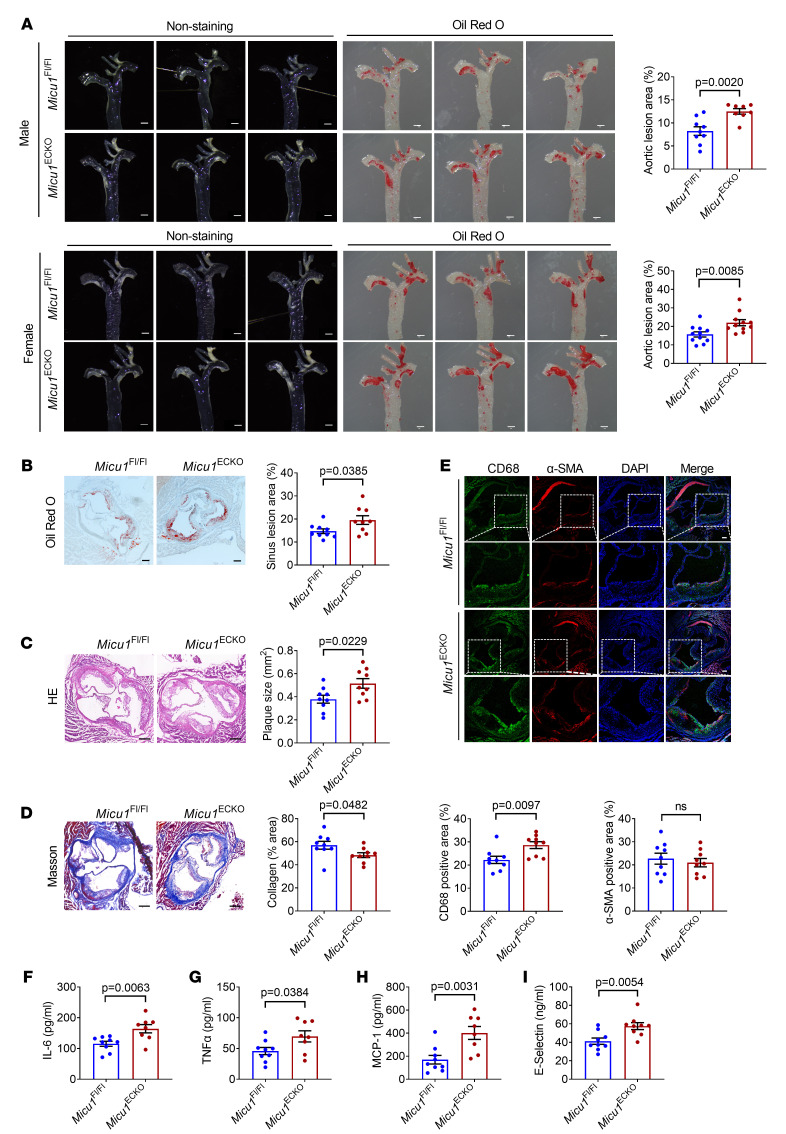
MICU1 deletion in ECs aggravates atherosclerosis. (**A**) Representative images of Oil Red O staining of atherosclerotic lesions of aorta in male (*n* = 8–9) and female (*n* = 11) *Micu1*^fl/fl^ mice or *Micu1*^ECKO^ mice infected with AAV8-PCSK9^D377Y^ after 12 weeks of Western diet feeding. Scale bars: 1 mm. (**B**–**D**) Oil Red O staining (**B**), H&E staining (**C**), or Masson staining (**D**) of lesions of the aortic root in male *Micu1*^fl/fl^ mice or *Micu1*^ECKO^ mice from **A** (*n* = 9). Scale bars: 200 μm. (**E**) Staining of CD68-positive macrophages in lesion area of the aortic sinus from male *Micu1*^fl/fl^ mice or *Micu1*^ECKO^ mice from **A** (*n* = 9). Scale bars: 100 μm. Original magnification, ×20. (**F**–**I**) ELISA of serum IL-6 (**F**), serum TNF-α (**G**), serum MCP-1 (**H**), and serum E-selectin (**I**) from male *Micu1*^fl/fl^ mice or *Micu1*^ECKO^ mice infected with AAV8-PCSK9^D377Y^ after 12 weeks of Western diet feeding (*n* = 8–9). Statistical analysis was performed by Student’s *t* test (**A**–**I**).

Lesion formation quantified by en face Oil Red O staining of the aortas was significantly increased in both male and female *Micu1*^ECKO^ mice compared with respective controls (Figure 6A). Furthermore, Oil Red O staining (Figure 6B) and histological analysis (Figure 6C) of the aortic sinus showed that deletion of endothelial *Micu1* increased the atherosclerotic plaque area. Masson staining of aortic root sections indicated that *Micu1* deficiency in endothelium reduced the proportion of collagen, a feature of plaque stability, in the aortic plaques (Figure 6D). The number of macrophages within the aortic sinus was increased in *Micu1*^ECKO^ mice compared with *Micu1*^fl/fl^ mice, whereas α-SMA expression showed no overt differences (Figure 6E). Similarly to the LPS-inflammation model, *Micu1*^ECKO^ mice also exhibited significantly elevated levels of serum IL-6, TNF-α, MCP-1, and E-selectin compared with *Micu1*^fl/fl^ mice (Figure 6, F–I). Thus, endothelial *Micu1* is a negative regulator of plaque growth and reduces features of plaque instability. The mechanism does not seem to involve alteration in circulating levels of lipoproteins, since no significant differences were observed between male *Micu1*^ECKO^ mice and *Micu1*^fl/fl^ mice, concerning serum triglyceride (TG), serum cholesterol (CHO), serum HDL, and serum LDL levels (Supplemental Figure 8, A–D).

Consistent with the phenotype obtained in male mice, *Micu1*^ECKO^ female mice also showed an increase of the aortic root and aortic sinus plaques, as well as infiltration of macrophages in the aortic sinus plaques compared with *Micu1*^fl/fl^ mice (Supplemental Figure 9, A, B, and D). *Micu1*^ECKO^ female mice also presented decreased collagen in the aortic sinus plaques (Supplemental Figure 9C). No significant differences were observed between *Micu1*^ECKO^ mice and control mice in terms of serum lipid profile (Supplemental Figure 9, E–H). There was an increase of serum IL-6 and TNF-α in *Micu1*^ECKO^ mice after 12 weeks of Western diet feeding (Supplemental Figure 9, I and J). Serum levels of MCP-1 and E-selectin did not change (Supplemental Figure 9, K and L).

To validate the potential role of the SIRT3/SOD2 pathway in *Micu1*-regulated atherosclerosis, we analyzed SIRT3 expression and levels of acetylated SOD2 in aortic root sections of hypercholesterolemic *Micu1*^ECKO^ and *Micu1*^fl/fl^ mice. *Micu1*^ECKO^ mice showed a decrease in SIRT3 expression and an increase in acetylated SOD2 (Ac-SOD2) levels compared with *Micu1*^fl/fl^ mice (Supplemental Figure 10), suggesting that *Micu1* enhances SIRT3 and reduces SOD2 acetylation. These results indicate that ablation of endothelial MICU1 promotes atherosclerosis partially through the SIRT3/SOD2 pathway.

### MICU1 overexpression in ECs attenuates atherosclerosis.

**Figure 7:**
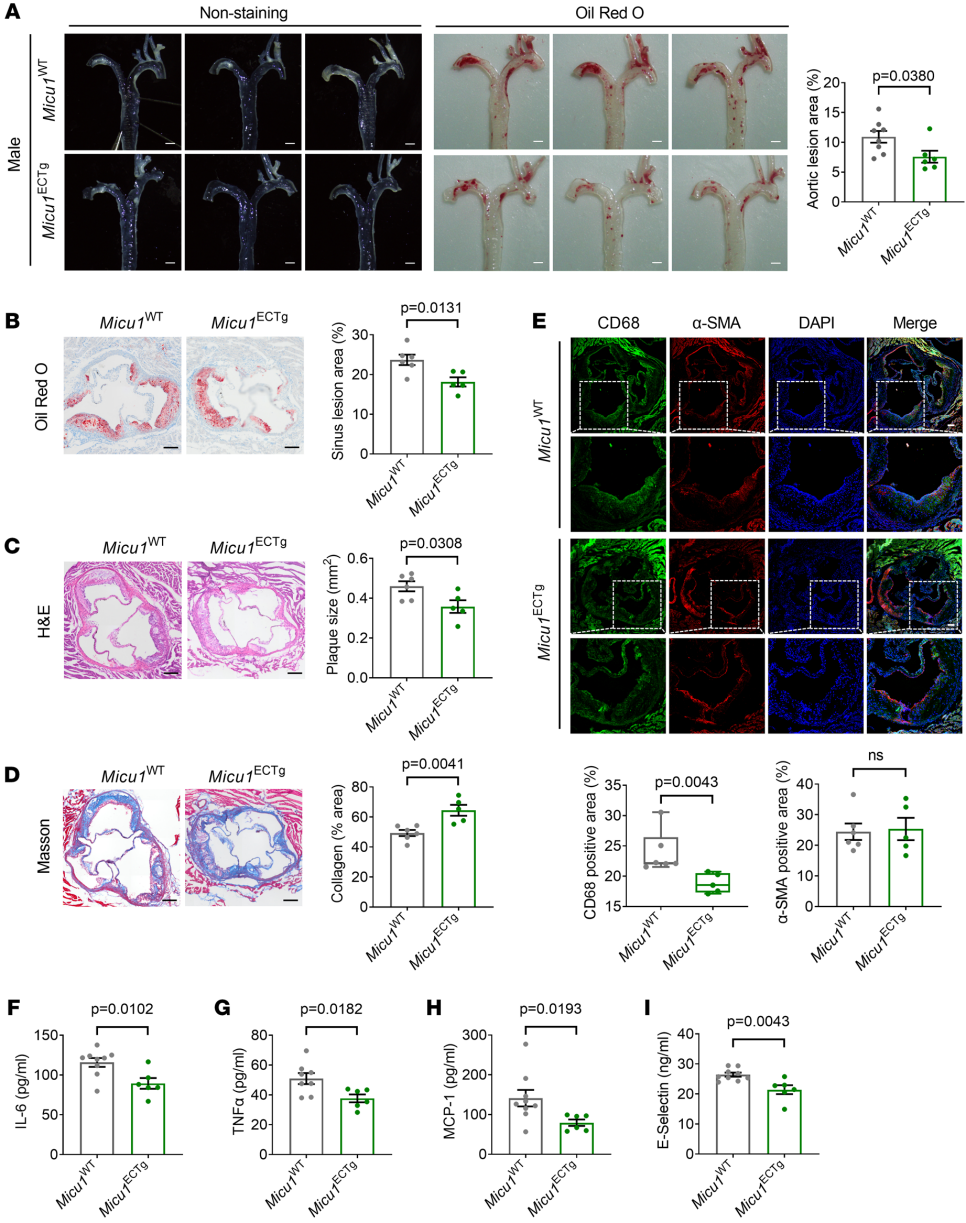
MICU1 overexpression in ECs attenuates atherosclerosis. (**A**) Representative images of Oil Red O staining of atherosclerotic lesions of aorta in male *Micu1*^WT^ mice or *Micu1*^ECTg^ mice infected with AAV8-PCSK9^D377Y^ after 12 weeks of Western diet (*n* = 6–8). Scale bars: 1 mm. (**B**–**D**) Oil Red O staining (**B**), H&E staining (**C**), or Masson staining (**D**) of lesions of the aortic root in male *Micu1*^WT^ mice or *Micu1*^ECTg^ mice from **A** (*n* = 5–6). Scale bars: 200 μm. (**E**) Staining of CD68-positive macrophages in lesion area of the aortic sinus from male *Micu1*^WT^ mice or *Micu1*^ECTg^ mice from **A** (*n* = 5–6). Scale bars: 100 μm. Original magnification, ×20. (**F**–**I**) ELISA of serum IL-6 (**F**), serum TNF-α (**G**), serum MCP-1 (**H**), and serum E-selectin (**I**) from male *Micu1*^WT^ mice or *Micu1*^ECTg^ mice infected with AAV8-PCSK9^D377Y^ after 12 weeks of Western diet (*n* = 6–9). Statistical analysis was performed by Student’s *t* test (**A**–**D**, α-SMA of **E**, **F**, **G**, and **I**), Mann-Whitney *U* test (CD68 of **E**), and Welch’s *t* test (**H**).

As a conditional gain-of-function approach, *Micu1*^ECTg^ mice were generated and plaque development was evaluated. EC-specific *Micu1* transgenic overexpression significantly decreased the plaque formation in en face aorta and aortic sinus compared with control mice (Figure 7, A–C). *Micu1*^ECTg^ mice also showed increased collagen (Figure 7D) and decreased macrophage infiltration in the aortic sinus (Figure 7E) compared with control mice. In addition, *Micu1*^ECTg^ mice presented decreased serum levels of IL-6, TNF-α, MCP-1, and E-selectin (Figure 7, F–I), but serum lipid profile showed no significant differences between *Micu1*^ECTg^ mice and control mice (Supplemental Figure 8, E–H). Collectively, these results suggest that endothelial *Micu1* limits vascular inflammation and atherosclerotic lesions in mice.

### Clinical relevance of MICU1 expression to cardiovascular diseases in patients.

**Figure 8:**
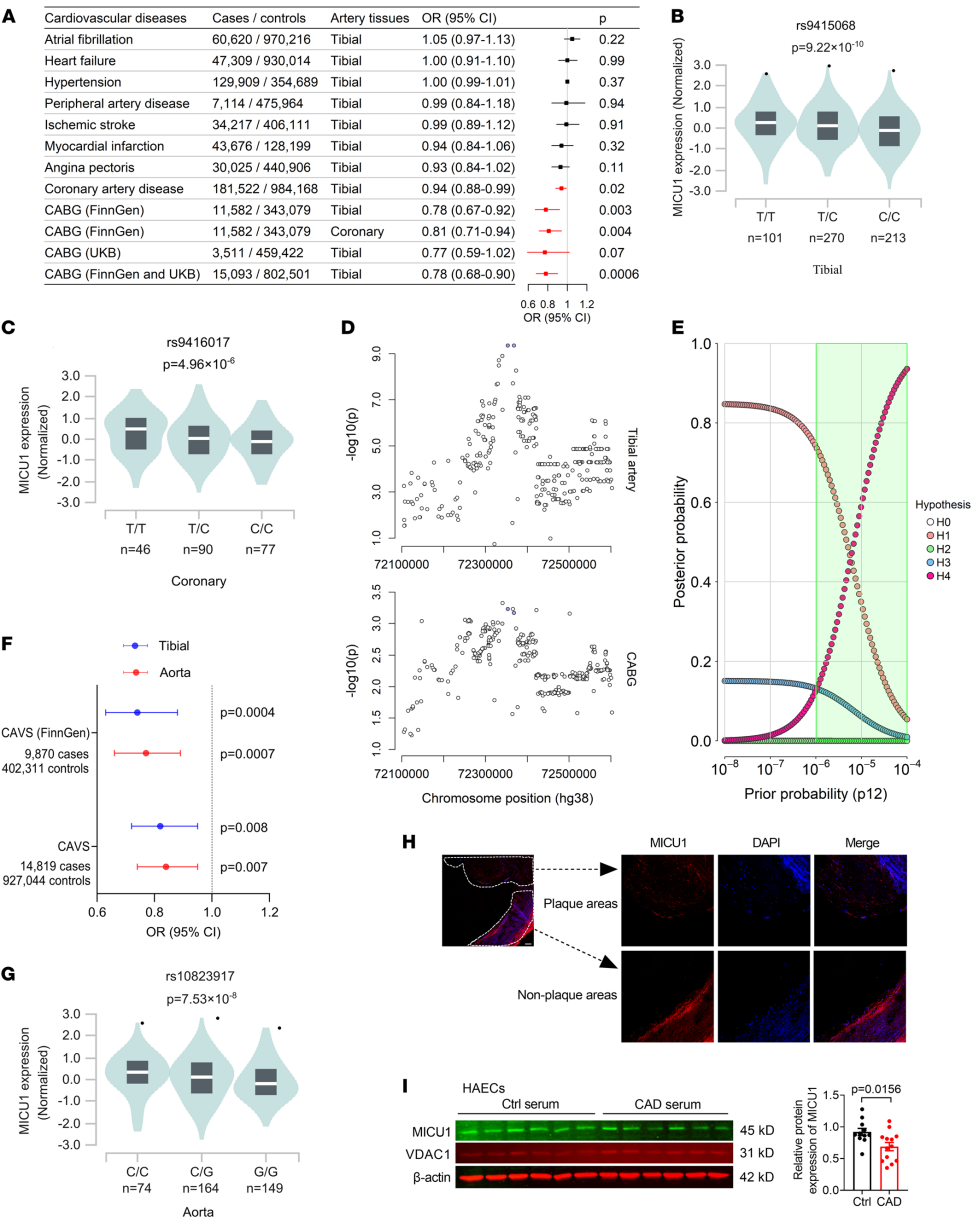
Clinical relevance of MICU1 expression to cardiovascular diseases in patients. (**A**) Two-sample Wald ratio MR testing effects of *MICU1* expression in vascular artery tissues on cardiovascular diseases. (**B**) eQTL analysis from GTEx revealed that the risk allele (C) associated with CABG is correlated with decreased *MICU1* expression in tibial artery tissue for a lead eQTL (rs9415068). (**C**) eQTL analysis from GTEx revealed that the risk allele (C) associated with CABG is correlated with decreased *MICU1* expression in coronary artery tissue for a lead eQTL (rs9416017). (**D**) Regional association plots highlighting ± 250 kb surrounding the lead eQTL (rs9415068) in *MICU1* locus for tibial artery (top) and CABG on chromosome 10. (**E**) Results from a Bayesian colocalization sensitivity analysis are presented. From the default (*P_12_* = 1 × 10^–5^) to more optimistic (*P_12_* = 1 × 10^–4^) priors, there is intermediate (59.3%) to strong (93.6%) posterior probability for a shared causal variant at the *MICU1* locus. The shaded green region denotes the range of prior probabilities, which results in probability of H4 (shared causal variant) > H3 (distinct causal variants). (**F**) Two-sample Wald ratio of MR testing effects of *MICU1* expression in vascular artery tissues on CAVS. (**G**) eQTL analysis revealed that the risk allele (G) associated with CAVS is correlated with decreased *MICU1* expression in aorta artery tissue for a lead eQTL (rs10823917). (**H**) Representative immunofluorescence is shown for the expression of MICU1 in human aortas with atherosclerosis. Confocal microscopy images showed MICU1 (red) and DAPI (blue). Scale bars: 100 μm (*n* = 4). (**I**) The expression of MICU1 in HAEC treatment with human serum of CAD compared with healthy condition for 24 hours (controls, *n* = 11; CAD, *n* = 13). Statistical analysis was performed by Student’s *t* test.

Finally, to explore the role of MICU1 in cardiovascular diseases in humans, we utilized Mendelian randomization (MR) studies. Interestingly, we detected significant associations between *MICU1* expression and CAD and CABG, but not with other cardiovascular events (Figure 8A). We constructed genetic instrumental variables (IVs) that represent changes in *MICU1* gene levels in blood vessels, using genetic variant associated with *MICU1* gene expression in tibial artery tissue sourced from GTEx. Two-sample Wald ratio MR revealed that increased expression of *MICU1* was marginally associated with decreased risk of CAD (odds ratio [OR] = 0.94, 95% CI, 0.88 to 0.99, *P* = 0.02) (Figure 8A). Notably, a more robust association was observed between *MICU1* expression and CABG (FinnGen) (OR = 0.78, 95% CI, 0.67 to 0.92, *P* = 0.003). The sensitivity analysis, which focused on coronary artery tissue, yielded similar results for CABG (OR = 0.81, 95% CI, 0.71 to 0.94, *P* =0.004). An independent MR analysis using CABG data in UK Biobank yielded a very similar effect size (OR = 0.77) with a marginal association (*P* = 0.07). We then combined the 2 CABG summary datasets to enhance the statistical power and obtain more precise estimations (OR = 0.78, 95% CI, 0.68 to 0.90, *P* = 0.0006). Consistently, analysis of expression quantitative trait loci (eQTL) data revealed that the risk allele (C), associated with CABG, correlates with decreased *MICU1* expression in both tibial and coronary artery tissues (Figure 8, B and C). To further validate MR findings, we conducted Bayesian colocalization analysis to assess the presence of a shared causal variant that influences both *MICU1* gene expression and CABG. We found intermediate-to-strong evidence supporting this shared causal variant (Figure 8, D and E). MR analysis also found a trend where increased expression of *MICU1* was weakly associated with a decreased risk of myocardial infarction (OR = 0.94) and angina pectoris (OR = 0.93) (Figure 8A), although these associations were not statistically significant.

In addition, we found that the genetic instrument (rs9415068) was associated with few phenotypes in phenome-wide association study (PheWAS) results when applying a conservative threshold of *P* = 3.56 × 10^–5^ (0.05/1403) in the UK Biobank and *P* = 2.08 × 10^–5^ (0.05/2408) in FinnGen. Interestingly, this variant was suggestively associated with operated calcific aortic valvular stenosis (CAVS) in the FinnGen (*P* = 0.0004). MR analysis suggested that increased expression of *MICU1* was associated with a decreased risk of CAVS, using both tibial (OR = 0.74, 95% CI, 0.63 to 0.88, *P* = 0.0004) and aorta artery eQTL data (OR = 0.77, 95% CI, 0.66 to 0.89, *P* = 0.0007) (Figure 8F). Using data from a recent large-scale summary statistic (29), MR analysis also yielded significant results (Figure 8F). eQTL data revealed that the risk allele (G) associated with CAVS also correlates with reduced *MICU1* expression in aorta artery tissue (Figure 8G).

To validate the correlation between MICU1 expression and cardiovascular disease, we collected atherosclerotic coronary arteries from patients with CAD and determined MICU1 levels in the plaque region. Interestingly, the expression of MICU1 was decreased in the intima layers of human arteries covering atherosclerotic plaques compared with the nonplaque region (Figure 8H). Besides, MICU1 protein levels were decreased in HAECs treated with serum from patients of CAD compared with that from healthy subjects (Figure 8I). These data indicate a possible role of MICU1 in atherosclerosis in humans.

## Discussion

MICU1 is a regulator of mitochondrial Ca^2+^ influx because of its EF-hand of Ca^2+^ binding sites and effect on MCU activity (11, 18). A previous study (30) has reported that MICU1 protein expression is decreased in ECs from patients with cardiovascular diseases, accompanied by increased [Ca^2+^]_m_ accumulation, mROS production, and halted EC migration. However, these effects were rescued by MICU1 overexpression. In addition, MICU1 protects against vascular leakage through regulating mitochondrial Ca^2+^ accumulation and oxidative burden (30). However, the precise role of endothelial MICU1 in vascular inflammation and atherosclerosis in vivo has not been reported. In the present study, we demonstrated that MICU1 is a crucial factor in suppressing endothelial inflammation and preventing the development of atherosclerosis. Specifically, endothelial *Micu1* deletion aggravated, while endothelial *Micu1* overexpression attenuated, vascular inflammation and atherosclerosis. Mechanistically, MICU1 gatekeeps mitochondrial Ca^2+^ homeostasis and prevents ROS production via the SIRT3/SOD2 pathway.

MICU1 is an important gatekeeper for mitochondrial Ca^2+^ influx and fitness. For example, Dong et al. reported that treatment of mice with perfluorooctane sulfonate (an organic pollutant) induced mitochondrial Ca^2+^ overload and promoted the assembly of the IP3R2-VDAC1-MICU1 complex in mouse liver (31). This discovery of VDAC1-MICU1 interaction provided an efficient pathway for mitochondrial Ca^2+^ uptake. In addition, Patel et al. reported that levels of mtCU components were altered in ECs under different shear-stress conditions, indicating a tight regulation of the mtCU by physiological and pathological hemodynamic forces (32). It is known that flow shear stress regulates focal development of atherosclerosis (33). MICU1 expression was decreased under both steady shear-stress and oscillatory shear-stress conditions. This is probably a consequence of multiple factors. In our study, we observed that conditional knockout or transgene of *Micu1* regulates atherosclerotic plaque development mainly in aortic arch and arterial branches, where blood flow pattern was oscillatory shear stress. It will be of interest to determine whether oscillatory shear stress can promote atherogenesis via disrupting MICU1-mediated mitochondrial homeostasis. Further, our findings of MICU1 levels decreased in ECs from humans with CAD are similar to the report by Hoffman et al. (30), which reported that MICU1 rescued ECs from excessive Ca^2+^ overload and oxidative stress, therefore inhibiting vascular leakage. MICU1 has also been found to attenuate cardiac microvascular injury of diabetic cardiomyopathy (34). The above evidence collectively suggests that MICU1 plays a key role in endothelial homeostasis and health.

Biologically, MICU1 regulates the level of mitochondrial Ca^2+^, the overload of which leads to mitochondrial dysfunction, elevated mROS, and bioenergetic disorders (reduced ATP production), culminating in multiple cardiovascular diseases including heart failure and myocardial ischemia/reperfusion injury (7, 8, 35, 36). Excessive accumulation of mitochondrial Ca^2+^ can also result in diabetes-associated cognitive impairment (37, 38). The levels of mROS are closely associated with inflammatory response (9, 10). The results were consistent with previous reports (17, 39). In this context, we demonstrate that *Micu1* depletion increased mitochondrial Ca^2+^ overload, mROS accumulation, and ATP depletion, indicative of mitochondrial dysfunction in ECs. This evidence provide insights into the regulatory mechanism of MICU1-mediated [Ca^2+^]_m_ homeostasis in preventing inflammation and atherogenesis.

SIRT3 is a protein deacetylase that is involved in various types of vascular pathologies (25). Decreased SIRT3 expression by cardiovascular risk factors is involved in disrupting mitochondrial homeostasis (25, 40–42). SIRT3 downregulation plays a causative role in vascular dysfunction and inflammatory response (25, 43, 44). It has been reported that excessive mitochondrial Ca^2+^ influx inhibited NAD^+^-dependent deacetylase activity of SIRT3, followed by a decrease in SOD2 activity, which contributes to aberrant ROS production and metastasis of hepatocellular carcinoma (26). In the present study, our data demonstrated that the downregulation of MICU1 reduced SIRT3 expression both in cultured ECs and atherosclerotic lesions in a mouse model. SIRT3, as a NAD^+^-dependent mitochondrial deacetylase, positively regulates SOD2 activity by deacetylation of specific lysine residues. Furthermore, SOD2-K68 acetylation is markedly increased in *Sirt3*-knockout mice, which is prohibited in *Sirt3* transgenic mice (25). This leads to the enhancement of endothelial permeability, activation of inflammasome pathways, and vascular inflammation. In addition, SOD2 deficiency in mice has already been proven to promote atherosclerotic lesion development in apolipoprotein E^–/–^ mice (45). Thus, we hypothesize that MICU1 regulates vascular inflammation and atherosclerosis potentially through the SIRT3/SOD2/mROS pathway.

Indeed, silencing of SIRT3 blocked the effect of MICU1 overexpression on SOD2 acetylation, mROS production, and endothelial inflammation. Mitochondrial Ca^2+^ overload attenuates SIRT3 deacetylase activity to inhibit the activity of SOD2, resulting in impaired mROS scavenging capacity. Dikalova et al. have reported that cardiovascular risk factors reduce the expression of SIRT3, thus contributing to SOD2 hyperacetylation, which leads to mROS generation and related vascular disorders, such as vascular inflammation and hypertension (25). In this study, we demonstrated the role of MICU1 in regulating SIRT3/SOD2 activity in the context of vascular inflammation and atherosclerosis. Intriguingly, Winnik et al. reported that *Sirt3* depletion does not affect atherosclerosis, but impairs rapid metabolic adaptation and increases systemic oxidative stress in low-density lipoprotein receptor knockout mice (46). This could be due to the fact that the global knockout of *Sirt3* may yield mixed results beyond regulating endothelial dysfunction. More recently, Cao et al. reported that *Sirt3*^ECKO^ mice exhibited enhanced plaque formation compared with control mice (47), which supports our finding that MICU1 deficiency promotes atherosclerosis partially through the SIRT3/SOD2 pathway. However, to elucidate the contributory role of SIRT3 to MICU1-mediated effects, rescue experiments involving SIRT3 manipulated mice might be necessary in future work.

### Clinical implications.

Overall, the strength of this study lies in its 2-pronged approach to elucidating the previously unrecognized role of MICU1 in regulating vascular inflammation and atherosclerosis by the combined use of experimental murine models of atherosclerosis and causal inference analysis of human genetic data. Our findings may carry important clinical implications. We observed reduced levels of MICU1 in the endothelial layer covering human atherosclerotic plaques and in primary HAECs exposed to serum from CAD patients. Additionally, alleles associated with increased risk of CAD and CABG also showed a correlation with decreased *MICU1* gene expression in arterial tissues. Consequently, diminished MICU1 levels could potentially serve as a diagnostic biomarker for the presence of atherosclerosis or as predictive markers for the likelihood of severe future events, such as CABG. This investigation has demonstrated that restoring MICU1 expression in ECs not only reduces vascular inflammation, but also helps prevent atherosclerosis. As such, therapies aimed at maintaining optimal levels of MICU1 in vascular ECs present an alternative approach for managing atherosclerosis. Given MICU1’s recognized role in regulating mitochondrial Ca^2+^ uptake, pharmacological targeting of MICU1 emerges as a viable strategy for preventing pathological mitochondrial Ca^2+^ overload in disease states. Recently, a range of compounds has been identified to inhibit mitochondrial Ca^2+^ uptake by targeting different components of the mtCU. Notably, MCU-i4 and MCU-i11 are 2 specific inhibitors that decrease mitochondrial Ca^2+^ uptake by binding to MICU1. MICU1 is essential for their inhibitory activity, making these compounds appealing candidates for MICU1-targeted drug development (48). The therapeutic potential of these pharmacological modulators in atherosclerosis remains to be explored.

In addition, our MR analysis indicated that increased expression of *MICU1* is associated with a decreased risk of CAVS. Currently, there are no medical therapies available to prevent CAVS or slow its progression. Notably, CAVS shares several features with atherosclerosis, including common risk factors, evidence of mitochondrial dysfunction, impaired oxidative stress responses, and inflammation (49). Based on these findings, we propose that therapeutic strategies effective against atherosclerosis by targeting of MICU1 might also be beneficial for CAVS. In sum, this study highlights MICU1 as a promising target for therapeutic intervention in cardiovascular diseases characterized by calcium overload and mitochondrial dysfunction.

### Study limitations.

We acknowledge that there are certain limitations in the present study. First, our experimental approach utilizes both tissue samples and murine models of atherosclerosis, which may not fully replicate all the features of the human condition, including potential differences in lipid metabolism. Second, the participants in our genetic study were predominantly of European descent. While this approach helps mitigate bias due to population stratification, it may limit the generalizability of our findings to other populations with varied genetic backgrounds. Third, based on our current knowledge, the genetic data on the exposure and outcome are taken from nonoverlapping datasets. The estimate from our 2-sample MR analysis is thus less biased, and any remaining bias is likely toward the null (50). Finally, although the results from population studies were generally consistent, some potential confounding effects or residual uncertainties may still persist. These issues could be further minimized in future analyses as larger and better-designed datasets become available.

In conclusion, our study highlights that MICU1 contributes to vascular resilience by preventing EC mitochondrial dysfunction, vascular inflammation, and atherosclerosis. This protective effect is achieved, at least in part, by limiting mitochondrial Ca^2+^ overload, which enhances the SIRT3/SOD2 pathway, thereby reducing excessive mROS and vascular inflammation. These findings underscore the potential to develop therapeutics targeting MICU1 and its downstream pathways, aiming to reduce residual cardiovascular risk and eventually the burden of ASCVD.

## Methods

### Sex as a biological variable.

Our study examined human samples of both male and female subjects. The mouse study also involved both male and female mice.

### Animal study.

Mice were randomized into each group by randomization table. *Micu1* flox mice (*Micu1*^fl/+^) and *Cdh5*-Cre mice were generated on a C57BL/6J background by Shanghai Model Organisms Center. EC-specific *Micu1* knockout mice (*Micu1*^ECKO^) were constructed by crossbreeding *Micu1*^fl/fl^ mice with *Cdh5*-Cre mice. EC-specific *Micu1* transgenic mice (*Micu1*^ECTg^) were obtained by crossbreeding Rosa26-CAG-LSL-Micu1-WPRE-polyA (*Rosa26*^LSL-Micu1^) mice (Shanghai Model Organisms) with *Cdh5*-Cre mice.

### Collection of human tissue samples.

Human tissues and serum were all collected from The First Affiliated Hospital of University of Science and Technology of China. Human aortic samples were obtained from patients with CAD who underwent aortic surgery. Serum samples of healthy people were from the physical examination center of The First Affiliated Hospital of University of Science and Technology of China, and serum samples of people with CAD were collected from vascular surgery (51).

### Statistics.

Statistical analyses were performed by GraphPad Prism 9.0 software or SPSS 24.0 software. All parametric data are presented as mean ± SEM. The data distribution was verified by Shapiro-Wilk normality test, and the homogeneity of variance was detected by the Brown-Forsythe test. For comparison between 2 groups, unpaired, 2-tailed Student’s *t* test was used for data of normal distribution with equal variances, the Welch’s *t* test was used for data of normal distribution but unequal variances, and the Mann-Whitney *U* test was used for data of nonnormal distribution. For groups of 3 or more, 1-way ANOVA followed by Bonferroni’s post hoc tests was used for data of normal distribution and the Kruskal-Wallis test followed by Dunn’s multiple comparisons test was used for data with nonnormal distribution. For grouped analysis, 2-way ANOVA followed by Bonferroni’s post hoc tests was used for data of normal distribution and multiple Mann-Whitney *U* tests were used for data of nonnormal distribution.

### Study approval.

The GWAS or eQTL summary data used in this work were obtained from publicly available datasets, which were approved by their original institutional ethics committees. All animal procedures used in this study were approved by the animal ethics committee of University of Science and Technology (approval no. USTCACUC212301048) and in accordance with the guidelines by the Institutional Animal Care and Use Committee. The collection of human aortic samples or serum were approved by the institutional review board (IRB) of The First Affiliated Hospital of University of Science and Technology (approval no. 2021KY-089; 2023KY-383; 2024KY-397). All procedures involved in this study were performed in accordance with the Declaration of Helsinki and in accordance with the relevant guidelines and regulations.

### Data availability.

The data for transcriptome analysis are available in the Genome Sequence Archive in the National Genomics Data Center, China National Center for Bioinformation / Beijing Institute of Genomics, Chinese Academy of Sciences (GSA-Human: HRA005700, https://ngdc.cncb.ac.cn/gsa-human). The values for all data points in the graphs are provided in the Supporting Data Values file. Additional information on materials is provided in Supplemental Tables 1–3.

Detailed procedures are provided in the Supplemental Methods.

## Author contributions

SX and JW conceived the project. LS, RL, ML, and MS performed experiments. QH, ZZ, ZL, ZW, SG, and YX assisted in study design and animal experiments. LS and RL performed data analysis. HJ collected human samples. LS, SX, and JW wrote the original draft of the manuscript. SX, JW, Y Huo, CLM, MB, LW, Y Huang, PCE, JP, GGC, BCB, SO, and JG reviewed and edited the manuscript. All authors have approved and reviewed the final manuscript.

## Supplementary Material

Supplemental data

Unedited blot and gel images

Supporting data values

## Version 1 04/01/2025

Electronic publication

## References

[1] Bjorkegren JLM, Lusis AJ (2022). Atherosclerosis: recent developments. Cell.

[2] Opstal TSJ (2020). Colchicine attenuates inflammation beyond the inflammasome in chronic coronary artery disease: a LoDoCo2 proteomic substudy. Circulation.

[3] Ridker PM (2023). Targeting residual inflammatory risk: The next frontier for atherosclerosis treatment and prevention. Vascul Pharmacol.

[4] Ridker PM (2023). Inflammation and atherosclerosis. Circulation.

[5] Xu S (2021). Endothelial dysfunction in atherosclerotic cardiovascular diseases and beyond: from mechanism to pharmacotherapies. Pharmacol Rev.

[6] Li X (2016). Mitochondrial reactive oxygen species mediate lysophosphatidylcholine-induced endothelial cell activation. Arterioscler Thromb Vasc Biol.

[7] Bertero E, Maack C (2018). Calcium signaling and reactive oxygen species in mitochondria. Circ Res.

[8] Garbincius JF, Elrod JW (2022). Mitochondrial calcium exchange in physiology and disease. Physiol Rev.

[9] Hall CJ (2018). Blocking fatty acid-fueled mROS production within macrophages alleviates acute gouty inflammation. J Clin Invest.

[10] Forrester SJ (2018). Reactive oxygen species in metabolic and inflammatory signaling. Circ Res.

[11] Perocchi F (2010). MICU1 encodes a mitochondrial EF hand protein required for Ca(2+) uptake. Nature.

[12] Baughman JM (2011). Integrative genomics identifies MCU as an essential component of the mitochondrial calcium uniporter. Nature.

[13] De Stefani D (2011). A forty-kilodalton protein of the inner membrane is the mitochondrial calcium uniporter. Nature.

[14] Kamer KJ, Mootha VK (2014). MICU1 and MICU2 play nonredundant roles in the regulation of the mitochondrial calcium uniporter. EMBO Rep.

[15] Raffaello A (2013). The mitochondrial calcium uniporter is a multimer that can include a dominant-negative pore-forming subunit. EMBO J.

[16] Sancak Y (2013). EMRE is an essential component of the mitochondrial calcium uniporter complex. Science.

[17] Mallilankaraman K (2012). MICU1 is an essential gatekeeper for MCU-mediated mitochondrial Ca(2+) uptake that regulates cell survival. Cell.

[18] Fan M (2020). Structure and mechanism of the mitochondrial Ca^2+^ uniporter holocomplex. Nature.

[19] Tsai CW (2022). Mechanisms and significance of tissue-specific MICU regulation of the mitochondrial calcium uniporter complex. Mol Cell.

[20] Csordas G (2013). MICU1 controls both the threshold and cooperative activation of the mitochondrial Ca²^+^ uniporter. Cell Metab.

[21] Antony AN (2016). MICU1 regulation of mitochondrial Ca(2+) uptake dictates survival and tissue regeneration. Nat Commun.

[22] Ali M (2022). MICU1-dependent mitochondrial calcium uptake regulates lung alveolar type 2 cell plasticity and lung regeneration. JCI Insight.

[23] Singh R (2022). Uncontrolled mitochondrial calcium uptake underlies the pathogenesis of neurodegeneration in MICU1-deficient mice and patients. Sci Adv.

[24] Murphy E, Liu J (2022). Mitochondrial calcium and reactive oxygen species in cardiovascular disease. Cardiovasc Res.

[25] Dikalova AE (2020). Mitochondrial deacetylase Sirt3 reduces vascular dysfunction and hypertension while Sirt3 depletion in essential hypertension is linked to vascular inflammation and oxidative stress. Circ Res.

[26] Ren T (2017). MCU-dependent mitochondrial Ca^2+^ inhibits NAD^+^/SIRT3/SOD2 pathway to promote ROS production and metastasis of HCC cells. Oncogene.

[27] Kobiyama K, Ley K (2018). Atherosclerosis. Circ Res.

[28] Xu S (2022). Vascular homeostasis in atherosclerosis: A holistic overview. Front Immunol.

[29] Thériault S (2024). Integrative genomic analyses identify candidate causal genes for calcific aortic valve stenosis involving tissue-specific regulation. Nat Commun.

[30] Hoffman Nicholas E (2013). MICU1 motifs define mitochondrial calcium uniporter binding and activity. Cell Rep.

[31] Dong Z (2022). Perfluorooctane sulfonate induces mitochondrial calcium overload and early hepatic insulin resistance via autophagy/detyrosinated alpha-tubulin-regulated IP3R2-VDAC1-MICU1 interaction. Sci Total Enviro.

[32] Patel A (2023). Modulation of the mitochondrial Ca^2+^ uniporter complex subunit expression by different shear stress patterns in vascular endothelial cells. Physiol Rep.

[33] Niu N (2019). Targeting mechanosensitive transcription factors in atherosclerosis. Trends Pharmacol Sci.

[34] Shi X (2023). Endothelial MICU1 alleviates diabetic cardiomyopathy by attenuating nitrative stress-mediated cardiac microvascular injury. Cardiovasc Diabetol.

[35] Santulli G (2015). Mitochondrial calcium overload is a key determinant in heart failure. Proc Natl Acad Sci U S A.

[36] Yang R (2022). Grpel2 alleviates myocardial ischemia/reperfusion injury by inhibiting MCU-mediated mitochondrial calcium overload. Biochem Biophys Res Commun.

[37] Li J (2023). Non-canonical function of DPP4 promotes cognitive impairment through ERp29-associated mitochondrial calcium overload in diabetes. iScience.

[38] Hui Y (2024). High glucose impairs cognitive function through inducing mitochondrial calcium overload in Treg cells. iScience.

[39] Dong Z (2017). Mitochondrial Ca^2+^ uniporter is a mitochondrial luminal redox sensor that augments MCU channel activity. Mol Cell.

[40] Freitas M (2015). Effects of aging and cardiovascular disease risk factors on the expression of sirtuins in the human corpus cavernosum. J Sex Med.

[41] Lai YC (2016). SIRT3-AMP-activated protein kinase activation by nitrite and metformin improves hyperglycemia and normalizes pulmonary hypertension associated with heart failure with preserved ejection fraction. Circulation.

[42] Jin L (2022). FGF21-Sirtuin 3 axis confers the protective effects of exercise against diabetic cardiomyopathy by governing mitochondrial integrity. Circulation.

[43] Palomer X (2020). SIRT3-mediated inhibition of FOS through histone H3 deacetylation prevents cardiac fibrosis and inflammation. Signal Transduct Target Ther.

[44] D’Onofrio N (2023). SIRT3 mediates the effects of PCSK9 inhibitors on inflammation, autophagy, and oxidative stress in endothelial cells. Theranostics.

[45] Ballinger SW (2002). Mitochondrial integrity and function in atherogenesis. Circulation.

[46] Winnik S (2014). Deletion of Sirt3 does not affect atherosclerosis but accelerates weight gain and impairs rapid metabolic adaptation in LDL receptor knockout mice: implications for cardiovascular risk factor development. Basic Res Cardiol.

[47] Cao X (2024). Role of Argininosuccinate Synthase 1 -dependent L-Arginine biosynthesis in the protective effect of endothelial Sirtuin 3 against atherosclerosis. Adv Sci (Weinh).

[48] Di Marco G (2020). A high-throughput screening identifies MICU1 targeting compounds. Cell Rep.

[49] Pedriali G (2020). Aortic valve stenosis and mitochondrial dysfunctions: clinical and molecular perspectives. Int J Mol Sci.

[50] Burgess S (2016). Bias due to participant overlap in two-sample Mendelian randomization. Genet Epidemiol.

[51] Su M (2025). Endothelial IGFBP6 suppresses vascular inflammation and atherosclerosis. Nat Cardiovasc Res.

