# Supplementary material for: Endothelial MICU1 protects against vascular inflammation and atherosclerosis by inhibiting mitochondrial calcium uptake
Source: J Clin Invest. 2025 Apr 1;135(7):e181928. doi: 10.1172/JCI181928 (PMC11957702; doi:10.1172/JCI181928)
Supplement: Supplemental data [file jci-135-181928-s283.pdf]

Supplemental Materials for

Endothelial MICU1 protects against vascular inflammation and atherosclerosis by inhibiting mitochondrial calcium uptake

Lu Sun, *et al*.

Author for correspondence:

Jianping Weng, MD, Email: wengjp@ustc.edu.cn

Suowen Xu, PhD, Email: sxu1984@ustc.edu.cn

The PDF file includes:

Supplemental Materials and Methods

Figures S1 to S10

Tables S1 to S3

Supplemental Materials and Methods

Animals

All animal procedures used in this study were approved by the animal ethics committee of University of Science and Technology of China (No. USTCACUC212301048) and in accordance with the guidelines stipulated by the Institutional Animal Care and Use Committee. Mice were housed in the temperature maintained at 22°C with a 12-h light/dark cycle. Mice were allowed free access to water and food unless otherwise indicated. Mice were randomized into each group by randomization table. Experiments involving LPS-induced inflammatory responses and *Micu1*^{ECTg} mice were performed in male mice. While, experiments involving *Micu1*^{ECKO} were performed in both male and female mice.

Generation of endothelial cell-specific *Micu1* knockout mice

Micu1 flox mice (*Micu1*$^{\mathrm{Fl/+}}$) and *Cdh5*-Cre mice (NM-KI-200173) were generated on C57BL/6J background by Shanghai Model Organisms Center (Shanghai, China). *Micu1* flox mice (*Micu1*$^{\mathrm{Fl/+}}$) were established by CRISPR/Cas9 technology at chromosome 10 in C57BL/6J background. Guide-RNA, Cas9 mRNA, and a vector containing donor DNA were injected into fertilized eggs by microinjection. Positive mice containing *Micu1* flanked by loxP sites (flox) were selected from offspring by sequencing. The Cre/LoxP strategy was used to generate endothelial cell-specific *Micu1* knockout mice (*Micu1*^{ECKO}). Briefly, *Micu1*$^{\mathrm{Fl/+}}$ mice were further intercrossed to obtain *Micu1*$^{\mathrm{Fl/Fl}}$ mice. *Micu1*^{ECKO} were constructed by crossbreeding *Micu1*$^{\mathrm{Fl/Fl}}$ mice with *Cdh5*-Cre mice, and *Micu1*$^{\mathrm{Fl/Fl}}$ mice were used for controls. The identification of *Micu1*^{ECKO} mice and *Micu1*$^{\mathrm{Fl/Fl}}$ mice were shown in Figure S2. En face immunofluorescent staining and intimal RNA from wild type and *Micu1*^{ECKO} mice was collected to validate the conditional knockout of *Micu1* in endothelial cells.

The blood pressure of the offspring was measured by the tail-cuff method with noninvasive blood pressure system (CODA Monitor, Kent Scientific). Mice were subjected to adaptive training and left unperturbed before measurement. The results of

at least three consecutive detections were performed to obtain average data for analysis.

Generation of endothelial cell-specific *Micu1* transgenic mice

Rosa26-CAG-LSL-Micu1-WPRE-polyA (*Rosa26*$^{\text{LSL-Micu1}}$) mice were generated on C57BL/6J background by Shanghai Model Organisms Center (Shanghai, China). CRISPR/Cas9 technology was used to obtain *Micu1* conditional expression mouse model of Rosa26 site-specific knock-in at chromosome 6 in C57BL/6J background. Guide RNA, Cas9 mRNA, targeting vector to recombination were injected to fertilized eggs by microinjection to obtain Rosa26-CAG-LSL-Micu1-WPRE-polyA (*Rosa26*$^{\text{LSL-Micu1}}$) mice. Endothelial cell-specific *Micu1* transgenic mice (*Micu1*^{ECTg}) were obtained by crossbreeding *Rosa26*$^{\text{LSL-Micu1}}$ mice with *Cdh5*-Cre mice. *Rosa26*$^{\text{LSL-Micu1}}$ mice labeled as *Micu1*^{WT} in this article were used for controls. The identification of *Micu1*^{ECTg} mice and *Micu1*^{WT} mice were shown in Figure S4.

Human tissue samples

The collection of human aortic samples and serum were approved by the institutional review board (IRB) of The First Affiliated Hospital of University of Science and Technology (No. 2021KY-089; 2023KY-383; 2024KY-397). All experiments were performed in accordance with the relevant guidelines and regulations. Human tissues and serum were all collected from The First Affiliated Hospital of University of Science and Technology of China. Patient tissue samples were obtained from the coronary arteries of heart transplant recipients with heart failure after myocardial infarction with informed consent (n=3). Serum samples of healthy subjects were from physical examination center of The First Affiliated Hospital of University of Science and Technology of China, and serum samples were also collected from patients with coronary artery disease (CAD). The detailed information of human serum was presented in Table S1.

Mendelian randomization

Two-sample Mendelian randomization (MR) using summary statistics was performed using the TwoSampleMR (1) package (version 0.5.7) in R (version 4.3.2). Genetic instruments were constructed using conditionally independent expression quantitative trait loci (eQTL) for MICU1 gene in vascular tissues from GTEx (version 8) (2, 3). The instrumental variable (IV) meeting a stringent threshold ($p < 5 \times 10^{-8}$) was found only in tibial artery tissue. We also evaluated the robustness of the MR findings in other arterial tissues, such as coronary artery tissue, using a more lenient threshold ($p < 5 \times 10^{-6}$) as part of our sensitivity analysis. Corresponding effects for selected IV on ischemic stroke (4), myocardial infarction (5), heart failure (6), angina pectoris (7), hypertension (8), peripheral artery disease (7) and atrial fibrillation (9) were obtained from the MRC IEU OpenGWAS database (https://gwas.mrcieu.ac.uk/) via the MR-Base platform (1).

For coronary artery disease (CAD) genetic analyses, we utilized summary statistics from the largest genome-wide association study (GWAS) meta-analysis to date, which comprised 181,522 cases of predominantly European ancestry (10). The genetic dataset of coronary artery bypass grafting (CABG) cases was obtained from the FinnGen study (R10) (11). We also obtained summary statistics for CABG from the UK Biobank (UKB) (12) through the MRC IEU OpenGWAS database (1). The UKB data analysis was done to validate the MR findings for CABG from the FinnGen study. We then employed an inverse-variance-weighted (IVW) fixed-effects model meta-analysis to combine the two CABG summary data for enhancing the statistical power. After harmonization of effect alleles, MR was performed using the Wald ratio method (when a single variant associated with gene expression was present) or the IVW method (when multiple genetic IVs associated with MICU1 expression were present). P values of less than 0.05 were considered significant.

Colocalization

Bayesian enumeration colocalization was performed as a sensitivity analysis for

CABG using the coloc package in R (version 4.3.2) (13, 14). Genetic variants ± 250 kb surrounding a lead eQTL in the *MICU1* locus were obtained from GTEx (version 8), with summary statistics deposited in the eQTL catalogue (2, 3). Corresponding CABG data for the analysis were also sourced from the aforementioned combined results of the FinnGen study (R10) and UKB (11, 12). Given the sensitivity of enumeration colocalization to the Bayesian priors specified, we explored a spectrum of priors that reflected different anticipated probabilities of a shared causal variant affecting both *MICU1* gene expression and CABG. We considered both the default prior ($p_{12} = 1 \times 10^{-5}$) and a range of more optimistic priors (15).

Look-up of phenome-wide associations

These were three lead IVs (rs9415068, rs9416017, and rs10823917), each corresponding to a different arterial tissue, exhibiting high linkage disequilibrium in Europeans ($r^2 > 0.65$). We focused our look-up of phenome-wide association studies (PheWAS) results on the most significant eQTL (rs9415068) to further explore its association with a broader range of phenotypes. We interrogated summary results from PheWAS analyzed using the UKB data (https://pheweb.org/UKB-SAIGE) (12). The PheWAS statistics were derived using imputed data from approximately 400,000 White British participants in the UKB. The ICD-based traits have been classified into 1,403 broad PheWAS codes. These statistics were computed using SAIGE, with adjustments made for genetic relatedness, sex, birth year, and the first 4 principal components. Additionally, the FinnGen data freeze 10 PheWeb browser (https://r10.finngen.fi/) (11), which includes association results for 2,408 endpoints derived from 412,181 Finnish biobank participants, was thoroughly looked up.

Cell culture and treatments

Different donors of human umbilical vein endothelial cells (HUVECs) were purchased from Lifeline Cell Technology (San Diego, CA, Cat# FC-0003, Lot# 08119/10128/04608). HUVECs were cultured in Endothelial Cell Medium (ScienCell,

Cat# 1001) consists of 500 ml of basal medium, 25 ml of fetal bovine serum (ScienCell, Cat# 0025), 5 ml of endothelial cell growth supplement (ScienCell, Cat# 1052) and 5 ml of antibiotic solution (penicillin /streptomycin, ScienCell, Cat# 0503).

Different donors of human aortic endothelial cells (HAECs) were purchased from Lifeline Cell Technology (Cat# FC-0014, Lot# 02357/01921/04675) and cultured in endothelial cell culture medium (Lifeline Cell Technology, Cat# LL-0013).

HUVECs or HAECs were 80% confluent before treatment with different stimuli including LPS (Sigma-Aldrich, Cat# L2630) or recombinant human TNFα (PeproTech, Cat# 300-01A). Cells were treated for indicated time with LPS (1 μg/ml) or TNFα (10 ng/ml) for monitoring inflammation, ROS production, mitochondrial respiration, and mitochondrial Ca^{2+} dynamics in HAECs.

Transcriptomic profiling

HUVECs were transfected with negative control siRNA or MICU1 siRNA (40 nM, RiboBio, Guangzhou, China) for 48 h. The sense sequence of MICU1 siRNA was (5'-3'): GCAGCUCAAGAAGCACUUCAA. Trizol (Invitrogen, Cat# 15596026) was used for RNA extraction. The strand-specific mRNA library building kit was used to generate mRNA libraries for high-throughput RNA sequencing using the BGISEQ-500 (BGI) platform (Gene Denovo, Guangzhou, China). The purified sequences were compared with the human genome from NCBI by hierarchical indexing for spliced alignment of transcripts (HISAT). Based on the pure sequences that met the quality control standards, the differentially expressed genes were screened according to the fold change (FC) ≥2 and false discovery rate (FDR) <0.05. The obtained differences were compared with the Gene Ontology (GO) database and Kyoto Encyclopedia of Genes and Genomes (KEGG) database to enrich gene functional attributes and pathways to clarify the effect of MICU1 downregulation on the overall biological function of endothelial cells. The raw sequence data have been deposited in the Genome Sequence Archive (Genomics, Proteomics & Bioinformatics 2021) in National Genomics Data Center (Nucleic Acids Res 2022), China National Center for

Bioinformation / Beijing Institute of Genomics, Chinese Academy of Sciences (GSA-Human: HRA005700, https://ngdc.cncb.ac.cn/gsa-human).

RNA extraction and quantitative RT-PCR

Total RNA was extracted from endothelial cells using Trizol (Invitrogen, Cat# 15596026). The collected RNA was converted to cDNA using a reverse transcription kit (TaKaRa, Japan, Cat# RR036A-1). Quantitative real-time-PCR (qRT-PCR) was performed with PCR System of LightCycler 96 (Roche) using TB Green Premix (TaKaRa, Japan, Cat# RR820) for amplification reaction. Relative expression of target gene was normalized to gene GAPDH. The sequences of primers used were listed in Table S2.

RT-PCR

Regular RT-PCR was performed using 2 × Taq Master Mix (Vazyme, China, Cat# P112-01) for genotyping of genetically modified mice. Amplification products amplified by ProFlex PCR system (Thermo Fisher Scientific) were separated in 1% agarose gel and analyzed by Gel Imaging System of JS-680D (P&Q Science Technology, China). The primer sequence used is as below:

Micu1$^{\mathrm{Fl/+}}$ primer sequence (5’-3’): P1, ACCCGACTAAAGAGCAGCTT (forward); P2, CCAGCTCAGAGGAGCACTAA (reverse).

Rosa26$^{\mathrm{LSL\text{-}Micu1}}$ primer sequence (5’-3’): P1, TCAGATTCTTTTATAGGGGACACA (forward); P2, TAAAGGCCACTCAATGCTCACTAA (reverse); P3, GTTGCGTCAGCAAACACAGT (forward); P4, ACTGTGATGGCAATGGGGAG (reverse).

Cdh5-Cre primer sequence (5’-3’): P1, CCAGGCTGACCAAGCTGAG (forward); P2, CCTGGCGATCCCTGAACA (reverse); P3, AGTGGCCTCTTCCAGAAATG (forward); P4, TGC GACTGTGTCTGATTTCC (reverse).

Western blot

Total protein was extracted using lysis buffer (Beyotime, China, Cat# P0013). The protein samples containing loading buffer (Beyotime, Cat# P0015) was subjected to SDS-PAGE electrophoresis. Then, the proteins were transferred to PVDF membrane (Millipore, Cat# IPVH00010). The blocking buffer (LI-COR, Cat# 927-60001) was used for blocking non-specific binding, and the primary antibodies were incubated overnight at 4℃. The information of primary antibodies was shown in Table S3. The following day, fluorescent secondary antibodies [IRDye® 800CW Goat anti-Rabbit IgG (LI-COR, Cat# 925-32211) or IRDye® 680RD Donkey anti-Mouse IgG (LI-COR, Cat# 926-68072)] was incubated. Images were detected by the Odyssey Infrared Imaging System (LI-COR) and analyzed by Image J software.

En face immunofluorescence staining

After sacrifice, mice were perfused with normal saline containing 40 U/ml heparin followed a slow drip of precooled 4% paraformaldehyde (PFA) solution for 15 min. The aorta was isolated and cleaned of peri-adventitial tissues and cut longitudinally, which were then penetrated in 0.1% Triton X-100 for 10 min, and blocked in 10% goat serum containing 2.5% Tween-20 for 1 h. The primary antibodies used include anti-MICU1 antibody (Sigma-Aldrich, Cat# HPA037480) and anti-VE-cadherin antibody (BD Biosciences, Cat# 555289). Antibodies were incubated overnight at 4℃. Samples were incubated with Alexa Fluor™ 488-conjugated secondary antibodies (Invitrogen, Cat# A11001) or Alexa Fluor™ 546-conjugated secondary antibodies (Invitrogen, Cat# A11010), and the nuclei were stained with DAPI (Beyotime, Cat# C1006) for 5 min. The images were detected by Leica STED high-resolution laser confocal system (Leica, Germany).

Evaluation of atherosclerosis

Male and female *Micu1*^{ECKO} mice, male *Micu1*^{ECTg} mice and respective littermate control mice about 8-week-old were administered via tail vein injection of adeno-associated virus expressing mouse recombinant PCSK9 with mutation of D377Y

(AAV8-PCSK9^{D377Y}, Vigene Biosciences, China) with 5×10^{11} viral particles in a 100 μL volume of sterile saline as previously described (16). Mice were fed with a western-type diet containing 1.25% cholesterol (Research Diets, Cat# D12108C) for 12 weeks. After that, mice were sacrificed according to animal protocol. Hearts and whole aortas were isolated and then fixed in 4% PFA solution. The whole aortas were subjected to Oil Red O (Poly Scientific R&D, Cat# s1849-16OZ) staining for quantification of atherosclerotic lesion using Image J software. The aortic root cryosections were cut to assess the lesion size in the aortic sinus. The sections were stained with Oil Red O solution and snapped by ZEISS Axio Imager (ZEISS, Germany). The positive area of Oil Red O staining was quantified by Image J software.

Measurements of lipid profile in mouse serum

Blood was collected retro-orbitally from experimental mice after overnight fasting. Then, the blood was centrifuged to obtain serum. Serum triglyceride (TG), cholesterol (CHO), high-density lipoprotein (HDL) and low-density lipoprotein (LDL) cholesterol levels were detected using automatic biochemical analyzer of Chemray (Rayto, Shenzhen, China).

Immunofluorescence staining and confocal microscopy

For immunofluorescence staining of sections of thoracic aorta or aortic sinus, the sections were permeabilized with 0.1% Triton X-100 at room temperature for approximately 15 min. Then, the sections were blocked with the immunostaining blocking solution (Beyotime, China, Cat# P0102) at room temperature for 1 h. After the blocking solution was discarded, the primary antibodies of VCAM1 (Abcam, Cat# ab134047), SIRT3 (Cell Signaling Technology, Cat# 5490), SOD2 (Cell Signaling Technology, Cat# 5490) and Ac-SOD2 (Abcam, Cat# ab137037) were added and placed at 4 °C overnight. The following steps were performed in the dark. The secondary fluorescent antibodies were added and incubated for 1 h, and then nuclei

were stained with DAPI staining solution (Beyotime, Cat# C1006) for 5 min. Images were examined using Leica STED high-resolution laser confocal system (Leica, Germany).

Histopathology

Frozen cross-sections from fixed aortic sinus were stained with hematoxylin and eosin (H&E, Servicebio, China, Cat# G1076), masson's trichrome (Servicebio, China, Cat# G1006) according to the manufacturer's protocols. Images were captured by ZEISS Axio Imager (ZEISS, Germany).

Enzyme-linked immunosorbent assay

The levels of cytokines in mouse serum were examined using enzyme-linked immunosorbent assay (ELISA) kits. Blood was collected retro-orbitally from mice. Then, the blood was centrifuged to obtain serum. ELISA of IL-6 (Boster, China, Cat# EK0411), TNFα (Boster, Cat# EK0527), MCP-1 (Boster, Cat# EK0568), CXCL-10 (Boster, Cat# EK0736) and E-selectin (Boster, Cat# EK0502) was performed according to the manufacturer's introductions. The results were obtained using multifunctional microplate reader of SpectraMax iD3 (Molecular Devices, USA).

siRNA transfection and adenovirus infection

Cells were seeded in dishes at a density of about 5×10^5/ml and then allowed to grow to about 60% cell density for experiments. Hiperfect transfection reagent (QIAGEN, Cat# 301705) was used to prepare small interfering RNA (RiboBio, Guangzhou, China) with final concentration of 20 nM, 40 nM or 80 nM. The Hiperfect transfection mixture was added to the dish in Opti-MEM medium (Gibco, Cat# 31985088), cultured for 6 h, then the solution was aspirated, and ECM medium was added to culture for 48 h. The efficiency of knockdown was detected by western blot.

Cells at 80% confluence were transfected MICU1 adenovirus (Vigene Biosciences, China) or control with same M.O.I. After 4-6 h of culture, the media was changed to

ECM complete medium for 48 h. MICU1 overexpression was detected by western blot.

Measurement of mitochondria Ca^{2+}

Mitochondrial-targeted calcium biosensors mitoGCAMP6s (17-19) from OBiO Technology (Shanghai, China) (Cat# H23762, Lot# DH0701) was used to monitor mitochondrial calcium uptake. Cells were transfected with mitoGCAMP6s adenovirus (Ad-mitoGCAMP6s) for 48 h. $[Ca^{2+}]_m$ was observed using a ZEISS laser confocal microscope LSM980 (ZEISS, Germany). After 1 min of baseline recording, histamine (HT, 50 μM, MedChemExpress, Cat# HY-B1204) was added. The fluorescence intensity of each group was monitored real time every 600 ms for 300 s.

Detection of mitochondrial ROS

For confocal microscopy, MitoSox (Invitrogen, Cat# M36008) fluorescent probe was used for detection of ROS levels in the mitochondria. The working solution diluted in HBSS with a final concentration of 5 μM, added to cultured cells, and incubated at 37 ℃ in the dark for 10 min. The fluorescence intensity was detected by ZEISS laser confocal microscope LSM980 (ZEISS, Germany) at 510/580 nm. For flow cytometry, after MitoSox incubation, cells were digested and placed in saline and examined by flow cytometry CytoFLEX (Beckman, USA).

Measurement of ROS

Dihydroethidium (DHE, Beyotime, Cat# S0063) fluorescent probe was used for detection of total ROS levels. The working solution of DHE was diluted to a final concentration of 1 μM, which was then added to cells and incubated at 37 °C in the dark for 30 min. The fluorescence intensity was detected by ZEISS laser confocal microscope LSM980 (ZEISS, Germany) at 510/580 nm.

Seahorse bioenergetics assay

Oxygen consumption rate (OCR) was measured using a Seahorse XFe96 analyzer (Agilent Technologies, Santa Clara, CA USA), following the manufacturer's protocol. HAECs were pretreated with siNC or siMICU1 for 24 h. Then, HAECs were seeded into XFe96 microplates (8,000 cells per well) and treated with vehicle or LPS (1 μg/ml, 16 h). Before testing, HAECs were equilibrated in Seahorse XF DMEM medium (Agilent, Cat# 103575-100), pH 7.4, without phenol red, supplemented with 1 mM pyruvate, 2 mM glutamine, and 10 mM glucose. HAECs were maintained in non-buffered assay medium in a non-CO_2 incubator for 1 h before the assay. OCR was measured using the Mito Stress Test Kit (Agilent, Cat# 103015-100) in the presence of oligomycin (1 μM), mitochondrial uncoupler carbonylcyano-4-(trifluoromethoxy) phenylhydrazone (FCCP, 1.5 μM), and respiratory chain inhibitors rotenone and antimycin A (0.5 μM)(20). The Seahorse XF-96 software was used to record and calculate OCR. Data were normalized to protein content in each treatment group using a BCA kit.

References

1. Hemani G, Zheng J, Elsworth B, Wade KH, Haberland V, Baird D, et al. The MR-Base platform supports systematic causal inference across the human phenome. *eLife.* 2018;7.
2. The GTEx Consortium atlas of genetic regulatory effects across human tissues. *Science (New York, NY).* 2020;369(6509):1318-30.
3. Kerimov N, Hayhurst JD, Peikova K, Manning JR, Walter P, Kolberg L, et al. A compendium of uniformly processed human gene expression and splicing quantitative trait loci. *Nature genetics.* 2021;53(9):1290-9.
4. Malik R, Chauhan G, Traylor M, Sargurupremraj M, Okada Y, Mishra A, et al. Multiancestry genome-wide association study of 520,000 subjects identifies 32 loci associated with stroke and stroke subtypes. *Nature genetics.* 2018;50(4):524-37.
5. Nikpay M, Goel A, Won HH, Hall LM, Willenborg C, Kanoni S, et al. A comprehensive 1,000 Genomes-based genome-wide association meta-analysis of coronary artery disease. *Nature genetics.* 2015;47(10):1121-30.
6. Shah S, Henry A, Roselli C, Lin H, Sveinbjörnsson G, Fatemifar G, et al. Genome-wide association and Mendelian randomisation analysis provide insights into the pathogenesis of heart failure. *Nature communications.* 2020;11(1):163.
7. Sakaue S, Kanai M, Tanigawa Y, Karjalainen J, Kurki M, Koshiba S, et al. A cross-population atlas of genetic associations for 220 human phenotypes. *Nature genetics.* 2021;53(10):1415-24.
8. Dönertaş HM, Fabian DK, Valenzuela MF, Partridge L, and Thornton JM. Common genetic associations between age-related diseases. *Nat Aging.* 2021;1(4):400-12.
9. Nielsen JB, Thorolfsdottir RB, Fritsche LG, Zhou W, Skov MW, Graham SE, et al. Biobank-driven genomic discovery yields new insight into atrial fibrillation biology. *Nature genetics.* 2018;50(9):1234-9.
10. Aragam KG, Jiang T, Goel A, Kanoni S, Wolford BN, Atri DS, et al. Discovery and systematic characterization of risk variants and genes for coronary artery disease in over a million participants. *Nat Genet.* 2022;54(12):1803-15.
11. Kurki MI, Karjalainen J, Palta P, Sipilä TP, Kristiansson K, Donner KM, et al. FinnGen provides genetic insights from a well-phenotyped isolated population. *Nature.* 2023;613(7944):508-18.
12. Sudlow C, Gallacher J, Allen N, Beral V, Burton P, Danesh J, et al. UK biobank: an open access resource for identifying the causes of a wide range of complex diseases of middle and old age. *PLoS Med.* 2015;12(3):e1001779.
13. Zuber V, Grinberg NF, Gill D, Manipur I, Slob EAW, Patel A, et al. Combining evidence from Mendelian randomization and colocalization: Review and comparison of approaches. *Am J Hum Genet.* 2022;109(5):767-82.
14. Wallace C. Eliciting priors and relaxing the single causal variant assumption in colocalisation analyses. *PLoS Genet.* 2020;16(4):e1008720.
15. Kim B, Zhao W, Tang SY, Levin MG, Ibrahim A, Yang Y, et al. Endothelial lipid droplets suppress eNOS to link high fat consumption to blood pressure elevation. *The Journal of clinical investigation.* 2023;133(24).
16. Bjørklund MM, Hollensen AK, Hagensen MK, Dagnaes-Hansen F, Christoffersen C, Mikkelsen JG, et al. Induction of atherosclerosis in mice and hamsters without germline

genetic engineering. *Circulation research.* 2014;114(11):1684-9.
17. Chen T-W, Wardill TJ, Sun Y, Pulver SR, Renninger SL, Baohan A, et al. Ultrasensitive fluorescent proteins for imaging neuronal activity. *Nature.* 2013;499(7458):295-300.
18. Mu D, Deng J, Liu KF, Wu ZY, Shi YF, Guo WM, et al. A central neural circuit for itch sensation. *Science (New York, NY).* 2017;357(6352):695-9.
19. Ashrafi G, de Juan-Sanz J, Farrell RJ, and Ryan TA. Molecular Tuning of the Axonal Mitochondrial Ca2+ Uniporter Ensures Metabolic Flexibility of Neurotransmission. *Neuron.* 2020;105(4):678-87.e5.
20. Luk C, Bridge KI, Warmke N, Simmons KJ, Drozd M, Moran A, et al. Paracrine role of endothelial IGF-1 receptor in depot-specific adipose tissue adaptation in male mice. *Nature communications.* 2025;16(1):170.

Supplemental Figures

Figure S1

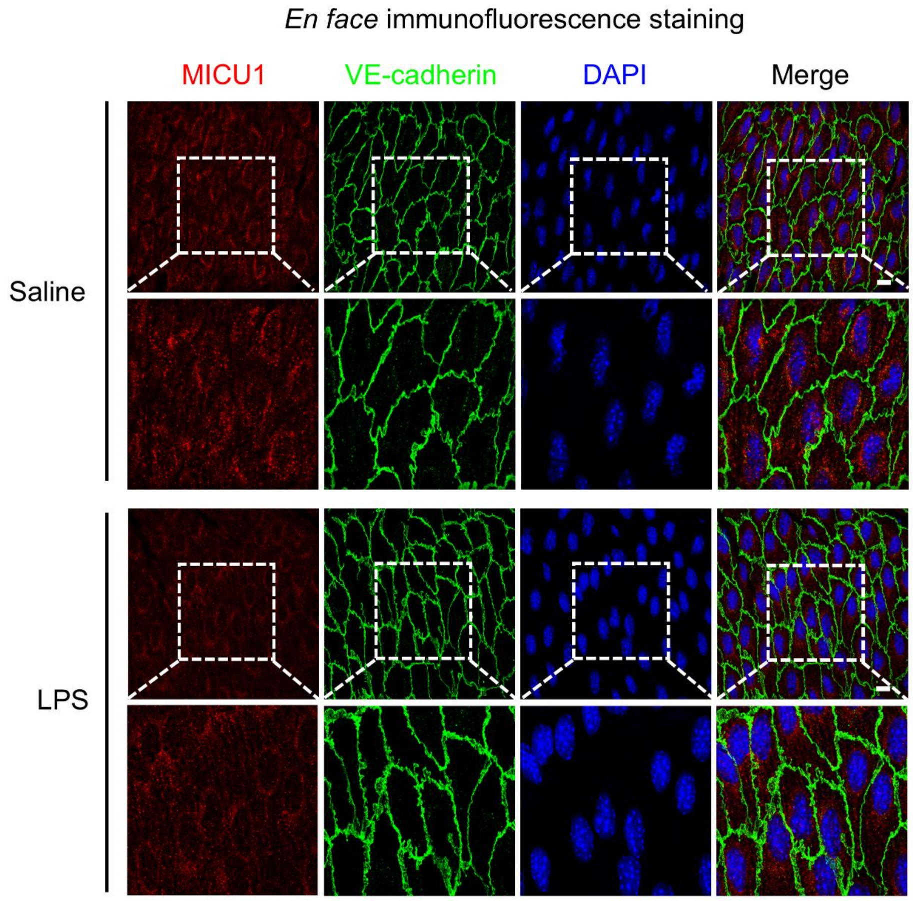

Figure S1. *En face* immunofluorescence staining of MICU1 in mouse aortic endothelium

Normal male C57BL/6J mice were intraperitoneally injected with LPS (10 mg/kg) for 6 h. *En face* staining was performed and images were captured using Confocal microscopy. MICU1 (red), VE-cadherin (green), and DAPI (blue). Scale bars, 10 μm (n=5).

Figure S2

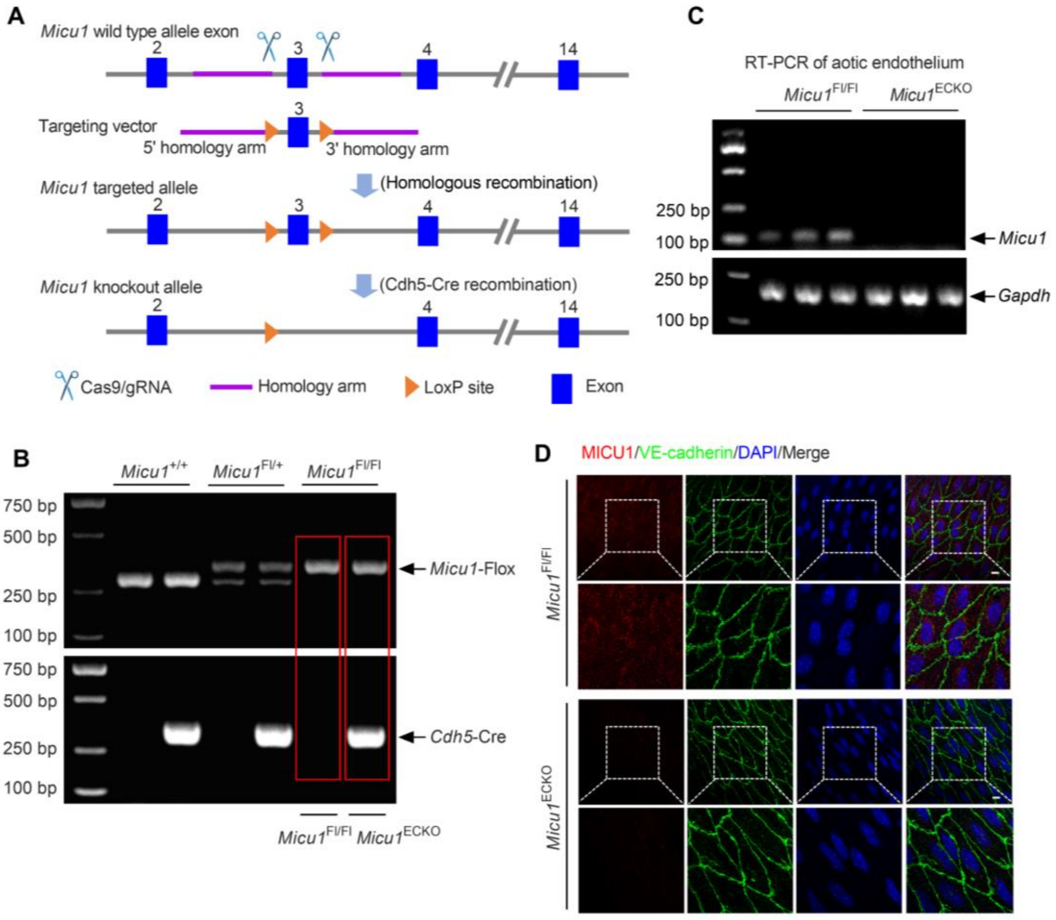

Figure S2. Generation and validation of *Micu1*$^{\mathbf{ECKO}}$ mice

(A) Strategy for generating endothelial-specific *Micu1* knockout (*Micu1*^{ECKO}) mice.

(B) Mouse genomic DNA were isolated for genotyping *Micu1*$^{Fl/Fl}$ mice and *Micu1*^{ECKO} mice.

(C) The mRNA level of MICU1 was measured by RT-PCR in mouse aortic endothelium lysate.

(D) *En face* immunofluorescence staining showed the protein expression of MICU1 was ablated in *Micu1*^{ECKO} mouse aorta (n=5). MICU1 (red), VE-cadherin (green), and DAPI (blue). Scale bars, 10 μm.

Figure S3

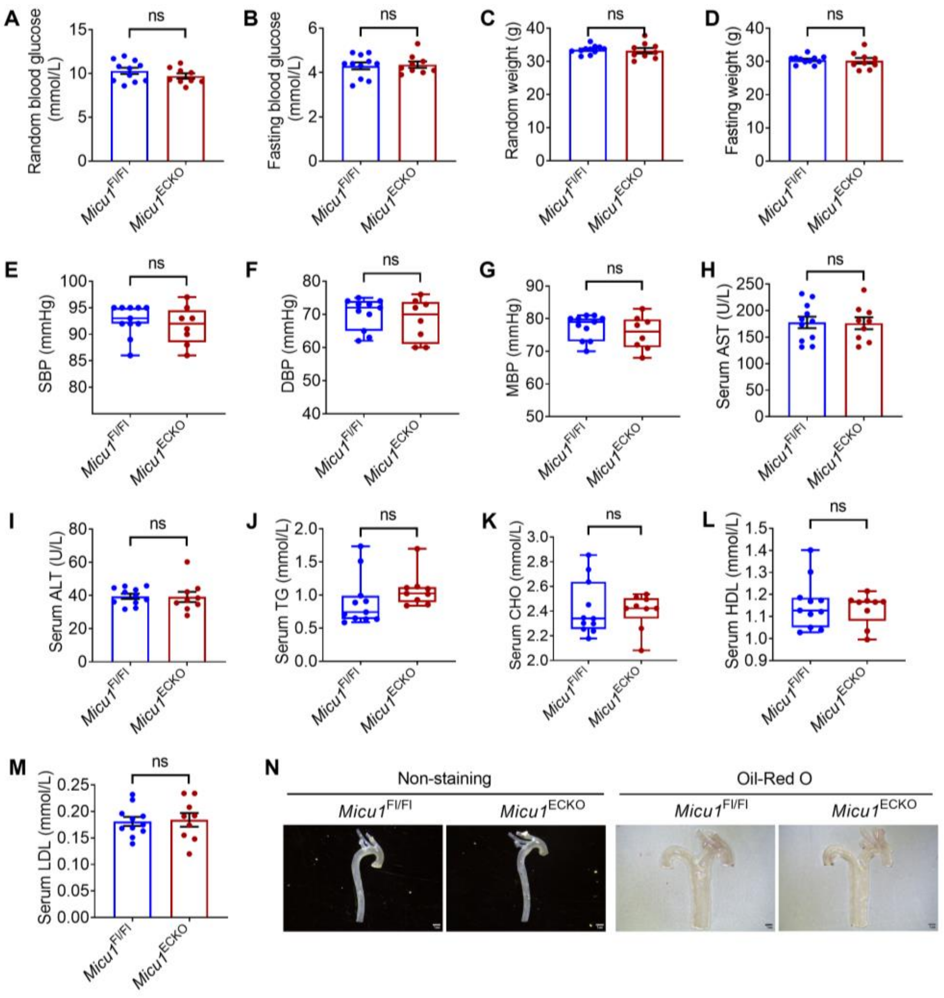

Figure S3. Baseline characterization of *Micu1*^{ECKO} mice

Blood glucose (A and B), body weight (C and D), systolic blood pressure (SBP, E), diastolic blood pressure (DPB, F), mean blood pressure (MBP, G), serum AST (H), serum ALT (I), levels of blood lipids (J-M) and morphology of *en face* aorta (N) from *Micu1*^{ECKO} mice and *Micu1*$^{\mathrm{Fl/Fl}}$ mice (n=9-11).

Statistical analysis was performed by Student t test (A-C, H, I, M), Welch's t test (D) and Mann-Whitney U test (E-G, J-L).

Figure S4

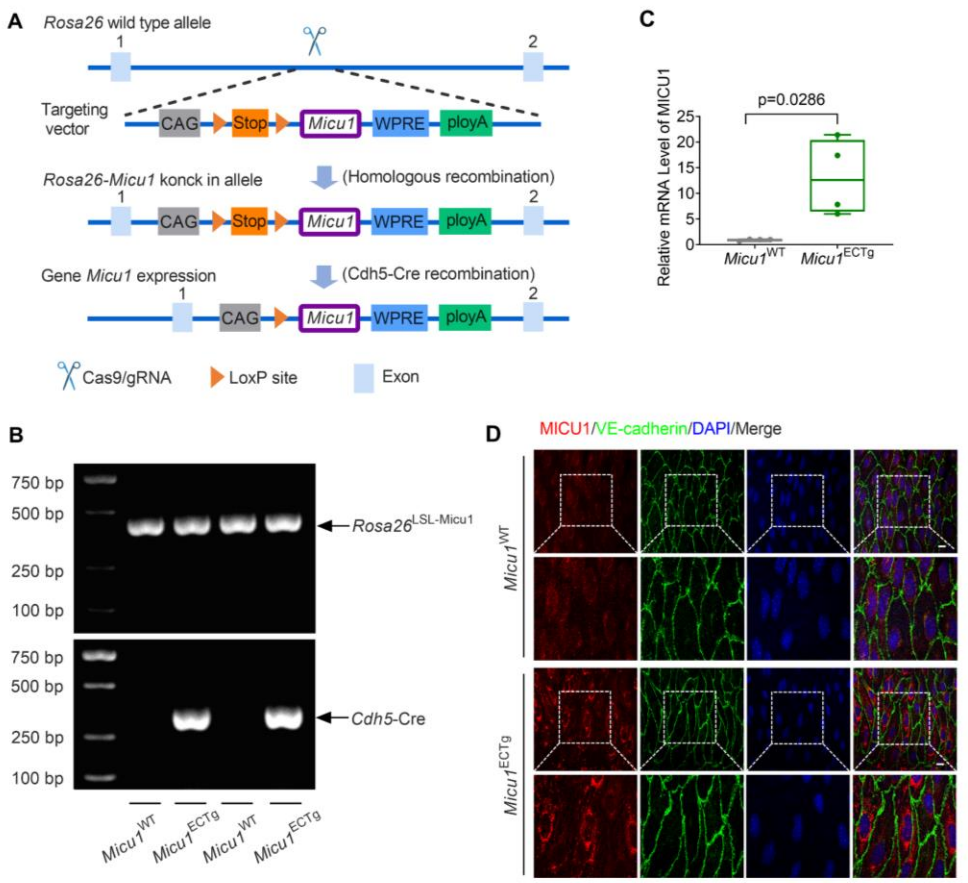

Figure S4. Generation and validation of *Micu1*^{ECTg} mice

(A) Strategy for generating endothelial-specific *Micu1* trangenic (*Micu1*^{ECTg}) mice.

(B) Mouse genomic DNA were isolated for genotyping *Micu1*^{WT} mice and *Micu1*^{ECTg} mice.

(C) The mRNA level of MICU1 was measured by quantitative RT-PCR in mouse aortic intimal lysate (n=4). Statistical analysis was performed by Student t test.

(D) *En face* immunofluorescence staining showed successful overexpression of MICU1 protein in *Micu1*^{ECTg} mouse aorta (n=5). MICU1 (red), VE-cadherin (green), and DAPI (blue). Scale bars, 10 μm.

Figure S5

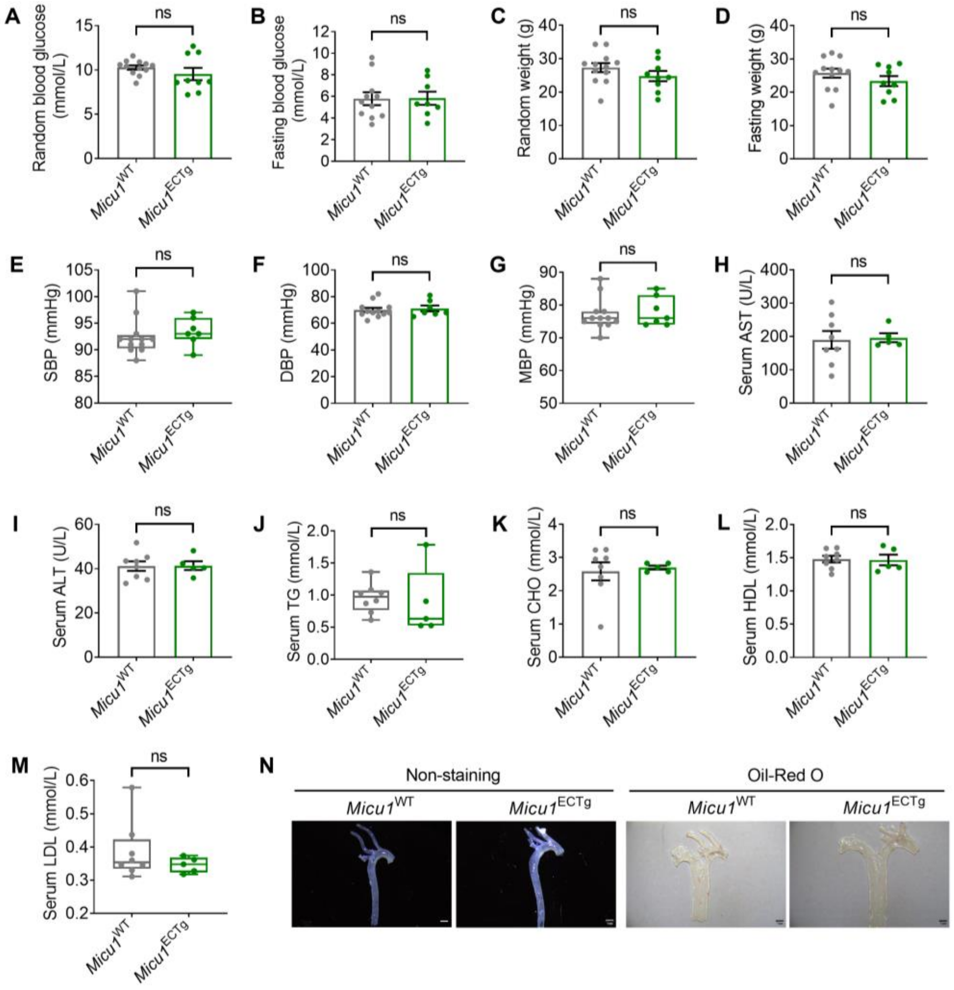

Figure S5. Baseline characterization of *Micu1*^{ECTg} mice

Blood glucose (A and B), body weight (C and D), SBP (E), DBP (F), MBP (G), serum AST (H), serum ALT (I), levels of blood lipids (J-M) and morphology of *en face* aorta (N) from *Micu1*^{ECTg} mice and *Micu1*^{WT} mice (n=5-12).

Statistical analysis was performed by Welch's t test (A, K), Student t test (B-D, F, H, I, L, K) and Mann-Whitney U test (E, G, J, M).

Figure S6

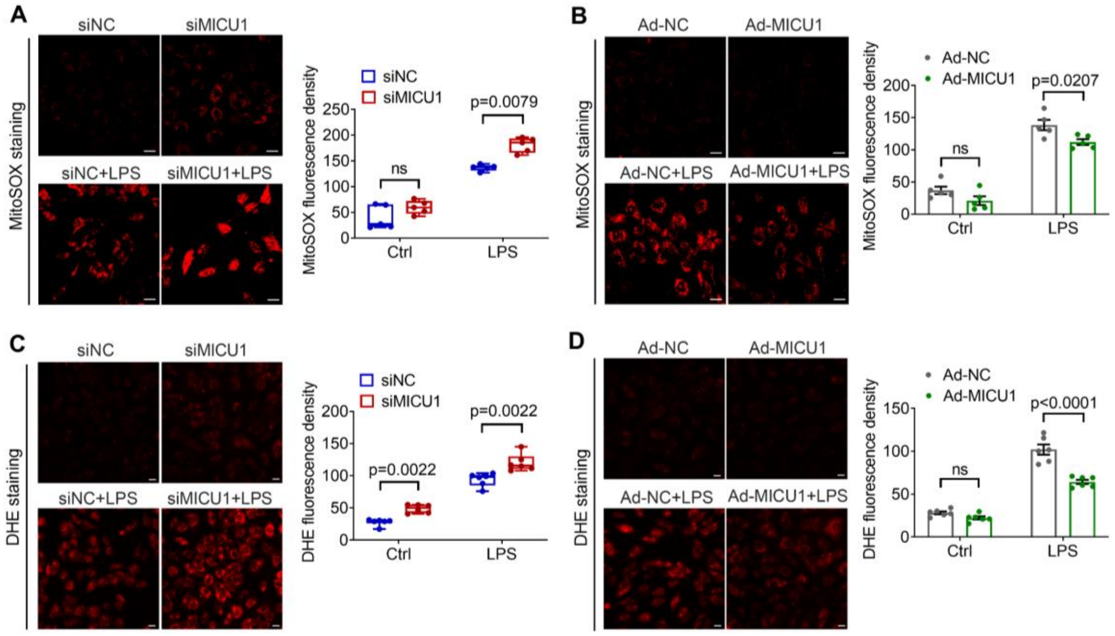

Figure S6. MICU1 modulates ROS levels in HUVECs

(A and B) Representative images showing mitoSOX fluorescence in HUVECs under the conditions of MICU1 silencing (A) or MICU1 overexpression (B) in the presence or absence of LPS (n=5). Scale bars, 20 μm.

(C and D) Representative images showing dihydroethidium (DHE) fluorescence in HUVECs under the conditions of MICU1 silencing (C) or MICU1 overexpression (D) in the presence or absence of LPS (n=6). Scale bars, 20 μm.

Statistical analysis was performed by multiple Mann-Whitney U tests (A and C) and 2-way ANOVA followed by Bonferroni post hoc tests (B and D).

Figure S7

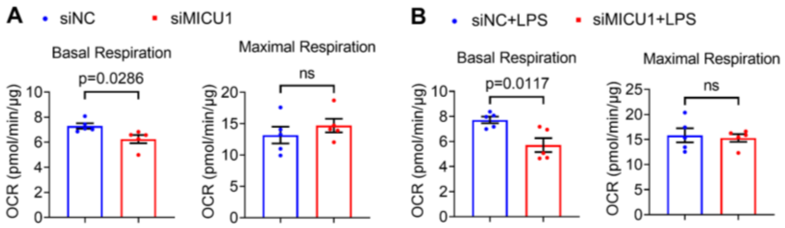

Figure S7. Quantification of mitochondria respiration in HAECs determined by Seahorse assay

Quantification and statistical analysis of basal respiration and maximal respiration in HAECs treated with siNC, siMICU1 with/without LPS (l μg/ml, 16 h) (n=4). Statistical analysis was performed by Student t test.

Figure S8

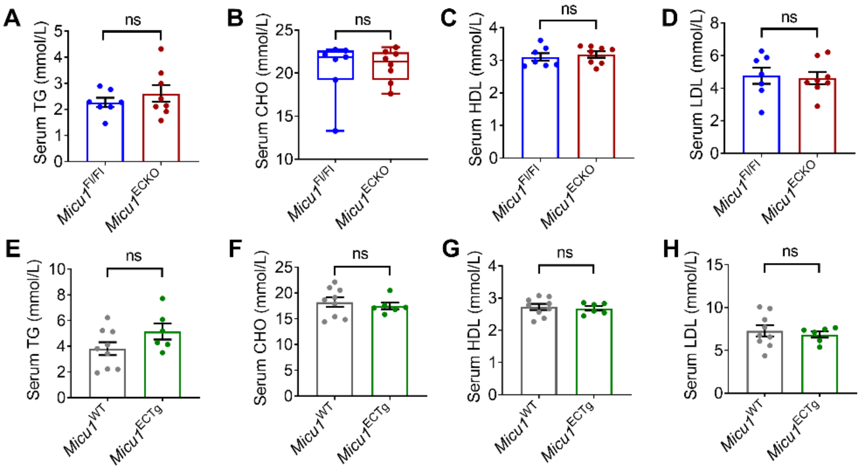

Figure S8. Lipid profile of *Micu1*^{ECKO} and *Micu1*^{ECTg} hypercholesterolemic mice compared with respective controls

(A-D) Serum levels of triglyceride (TG, A), total cholesterol (CHO, B), high-density lipoprotein (HDL, C) and low-density lipoprotein (LDL, D) were detected in male *Micu1*$^{Fl/Fl}$ mice or *Micu1*^{ECKO} mice infected with AAV8-PCSK9^{D377Y} after 12 weeks of western-type diet feeding (n=7-8).

(E-H) TG (E), CHO (F), HDL (G) and LDL (H) were detected in male *Micu1*^{WT} mice or *Micu1*^{ECTg} mice infected with AAV8-PCSK9^{D377Y} after 12 weeks of Western-type diet feeding (n=6-9).

Statistical analysis was performed by Student t test (A, C-H) and Mann-Whitney U test (B).

Figure S9

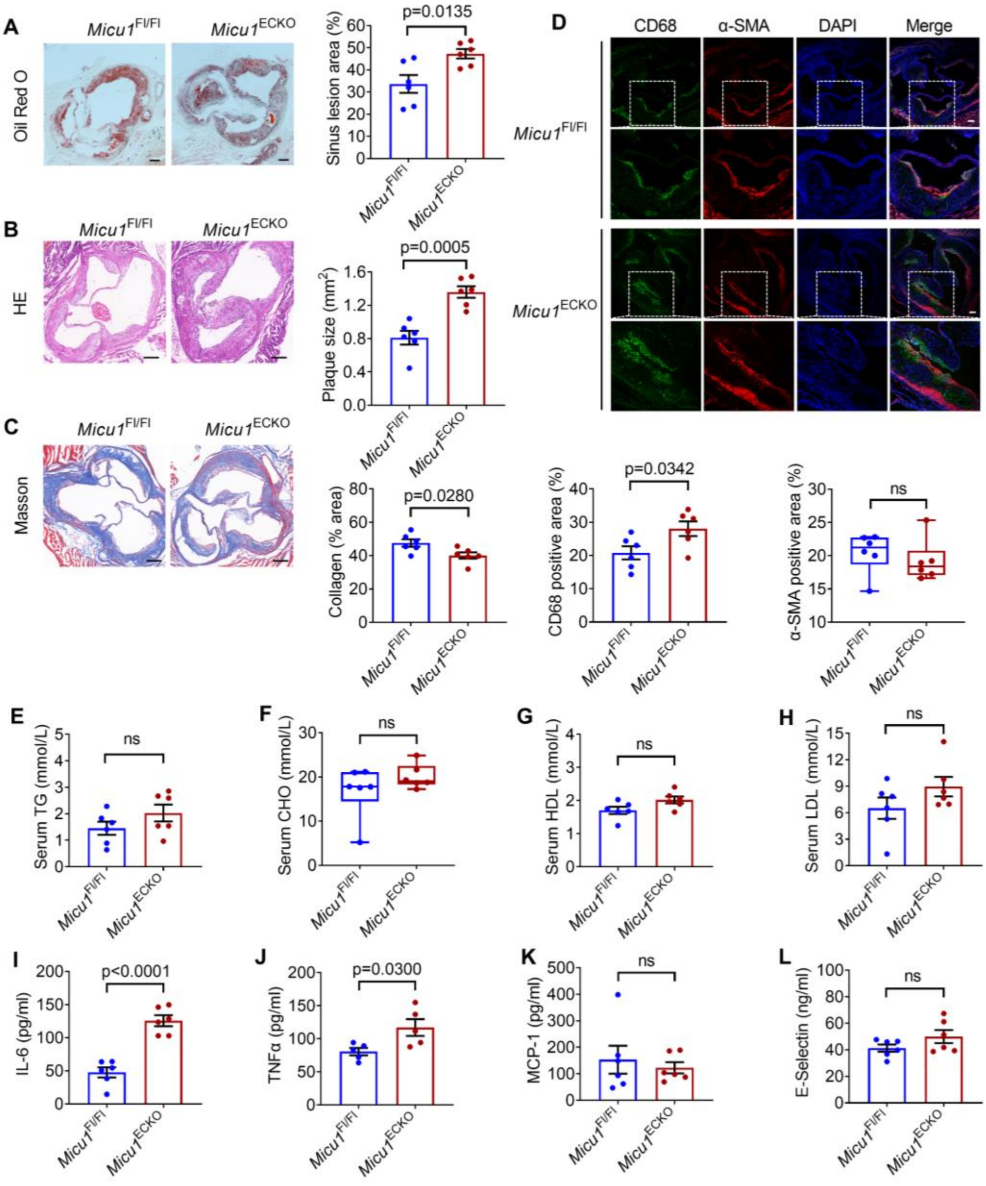

Figure S9. *Micu1* deletion in EC promoting Western diet-induced atherosclerosis in female mice

(A) Representative images of Oil Red O staining of aortic sinus from female *Micu1*$^{Fl/Fl}$ mice or *Micu1*^{ECKO} mice infected with AAV8-PCSK9^{D377Y} after 12 weeks of Western-type diet feeding (n=6). Scale bars, 1 mm.

(B and C) H&E (B) and Masson staining (C) of lesions of the aortic root in female *Micu1*$^{Fl/Fl}$ mice or *Micu1*^{ECKO} mice (n=6). Scale bars, 200 μm.

(D) Staining of CD68-positive macrophages in aortic sinus from female *Micu1*$^{Fl/Fl}$

mice or *Micu1*^{ECKO} mice (n=6). Scale bars, 100 μm.

(E-H) Serum levels of TG, CHO, HDL and LDL were detected in female *Micu1*$^{Fl/Fl}$ mice or *Micu1*^{ECKO} mice infected with AAV8-PCSK9^{D377Y} after 12 weeks of western diet (n=6).

(I-L) ELISA of serum IL-6 (I, n=6), TNFα (J, n=5), MCP-1 (K, n=6), E-selectin (L, n=6) in female *Micu1*$^{Fl/Fl}$ mice or *Micu1*^{ECKO} mice infected with AAV8-PCSK9^{D377Y} after 12 weeks of Western diet.

Statistical analysis was performed by Student t test (A-C, CD68 of D, E, G-L) and Mann-Whitney U test (α-SMA of D, F).

Figure S10

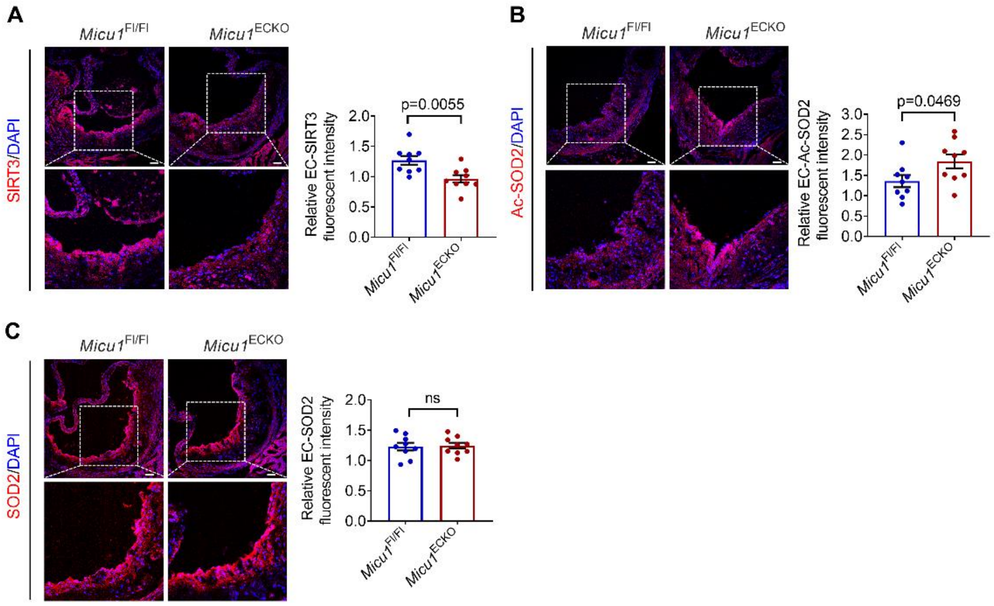

Figure S10. Immunofluorescence staining of SIRT3, Ac-SOD2 and SOD2

(A) SIRT3, (B) Ac-SOD2, (C) SOD2 protein expression in aortic sinus cryosections from male *Micu1*$^{Fl/Fl}$ mice or *Micu1*^{ECKO} mice infected with AAV8-PCSK9^{D377Y} after 12 weeks of Western-type diet feeding (n=9). Scale bars, 50 μm.

Statistical analysis was performed by Student t test (A-C).

Supplemental Tables

Table S1. Demographic summary of CAD patients and control subjects

Parameter	CAD (n=13)	Controls (n=11)	p Value
Male/female	12/1	8/3	0.3002
Age, y	66.9±11.7	40.2±15.8	<0.0001
CAD, n (%)	13 (100)	0 (0)	N/A
Hypertension, n (%)	10 (76.9)	1 (9.1)	0.0013
Diabetes, n (%)	7 (53.8)	0 (0)	0.0059
TC (mmol/L)	3.87±0.90	5.27±0.85	0.0008
TG (mmol/L)	1.32±0.82	2.54±2.99	0.1711
HDL (mmol/L)	0.99±0.36	1.41±0.28	0.0047
LDL (mmol/L)	2.38±0.70	3.16±0.51	0.0056

CAD, coronary artery disease; N/A, not applicable; TC, total cholesterol; TG, triglyceride; HDL, high-density lipoprotein; LDL, low-density lipoprotein.

Table S2. List of primers for qRT-PCR

Description	Species	Sequence (5'-3')
Micu1	Mouse	forward: CTTGAATGGAGACGGAGAGG<br>reverse: ACTTGAGGGTGTTCCCAGTG
Gapdh	Mouse	forward: GGTTGTCTCCTGCGACTTCA<br>reverse: TGGTCCAGGGTTTCTTACTCC
MICU1	Human	forward: GTGTTCAGCCCTCACAACCT<br>reverse: TCTCCCATCCACAGGGTCAT
CXCL1	Human	forward:CCCAAGAACATCCAAAGTGTGA<br>reverse: CAAGCTTTCCGCCCATTCT
CXCL2	Human	forward: CCTGCAGGGAATTCACCTCA<br>reverse: TGAGACAAGCTTTCTGCCCA
CXCL3	Human	forward: GTGAATGTAAGGTCCCCCGG<br>reverse: TTCTGAACCATGGGGGATGC
CX3CL1	Human	forward: GGGCGTCCTTATCACTCCTG<br>reverse: TGGTAGGTGAACATGGCCAC

IL12A	Human	forward: AGTTTGGCCAGAAACCTCCC<br>reverse: TTTGTCTGGCCTTCTGGAGC
IL15	Human	forward: GTTTCAGTGCAGGGCTTCCT<br>reverse: TGCAACTGGGGTGAACATCA
CCL5	Human	forward: GTGTGCCAACCCAGAGAAGA<br>reverse: GCCTCCCAAGCTAGGACAAG
VEGF-C	Human	forward: GCAGTTACGGTCTGTGTCCA<br>reverse: TCCTTGAGTTGAGGTTGGCC
MYD88	Human	forward: ACTTGGAGATCCGGCAACTG<br>reverse: TTGGTAAGCAGCTCGAGCAG
CSF1	Human	forward: CCAGCAACTTCCTCTCAGCA<br>reverse: GGGGTGTGATTCCAGTCCTG
IFNβ1	Human	forward: GCCGCATTGACCATCTATGA<br>reverse: GCCAGGAGGTTCTCAACAATAG
IL6	Human	forward: TGCAATAACCACCCCTGACC<br>reverse: AGCTGCGCAGAATGAGATGA
CXCL10	Human	forward: TGTACGCTGTACCTGCATCA<br>reverse: GCAATGATCTCAACACGTGGA
VCAM1	Human	forward: TGTCAATGTTGCCCCCAGA<br>reverse: TGCTCCACAGGATTTTCGGA
TNFα	Human	forward: GCACTGAAAGCATGATCCGG<br>reverse: AGAGGCTGAGGAACAAGCAC
MCP1	Human	forward: TGTCCCAAAGAAGCTGTGATC<br>reverse: ATTCTTGGGTTGTGGAGTGAG
GAPDH	Human	forward: CACCATCTTCCAGGAGCGAG<br>reverse: CCTTCTCCATGGTGGTGAAGAC

Table S3. List of antibodies for immunoblotting and immunofluorescence

**Antibodies for western blot**				
**Name**	**Source**	**Cat. No.**	**Species**	**Titer**
MICU1	Sigma-Aldrich	HPA037480	Rabbit	1:500
VDAC1	Santa Cruz	sc-390996	Mouse	1:250

	Biotechnology			
β-actin	Proteintech	66009-1-Ig	Mouse	1:5000
SIRT3	Cell Signaling Technology	5490	Rabbit	1:1000
VCAM1	Abcam	ab134047	Rabbit	1:1000
Ac-SOD2	Abcam	ab137037	Rabbit	1:800
SOD2	Cell Signaling Technology	13141	Rabbit	1:1000
GAPDH	Proteintech	60004-1-Ig	Mouse	1:5000
IRDye® Goat anti-Rabbit IgG	LI-COR	925-32211	Goat	1:10000
IRDye® Donkey anti-Mouse IgG	LI-COR	926-68072	Donkey	1:10000
**Antibodies for immunofluorescence**				
**Name**	**Source**	**Cat. No.**	**Species**	**Titer**
MICU1	Sigma-Aldrich	HPA037480	Rabbit	1:50
VE-cadherin	BD Biosciences	555289	Rat	1:100
ICAM1	Abcam	ab222736	Rabbit	1:100
VCAM1	Abcam	ab134047	Rabbit	1:100
CD68	Bio-Rad	MCA1957GA	Rat	1:100
α-SMA	Proteintech	14395-1-AP	Rabbit	1:100
SIRT3	Cell Signaling Technology	5490	Rabbit	1:100
Ac-SOD2	Abcam	ab137037	Rabbit	1:50
SOD2	Proteintech	24127-1-AP	Rabbit	1:100
Alexa Fluor™ 488 Goat anti-Rabbit IgG	Beyotime	A0423	Goat	1:1000
Alexa Fluor™ 488 Goat anti-Mouse IgG	Invitrogen	A-11001	Goat	1:1000
Alexa Fluor™ 546 Goat anti-Rabbit IgG	Invitrogen	A-11010	Goat	1:1000
Alexa Fluor™ 488 Goat anti-Rat IgG	Invitrogen	A-11006	Goat	1:1000
