# Supplementary figures and images for: Endothelial MICU1 protects against vascular inflammation and atherosclerosis by inhibiting mitochondrial calcium uptake
Source: J Clin Invest. 2025 Apr 1;135(7):e181928. doi: 10.1172/JCI181928 (PMC11957702; doi:10.1172/JCI181928)

F2-K

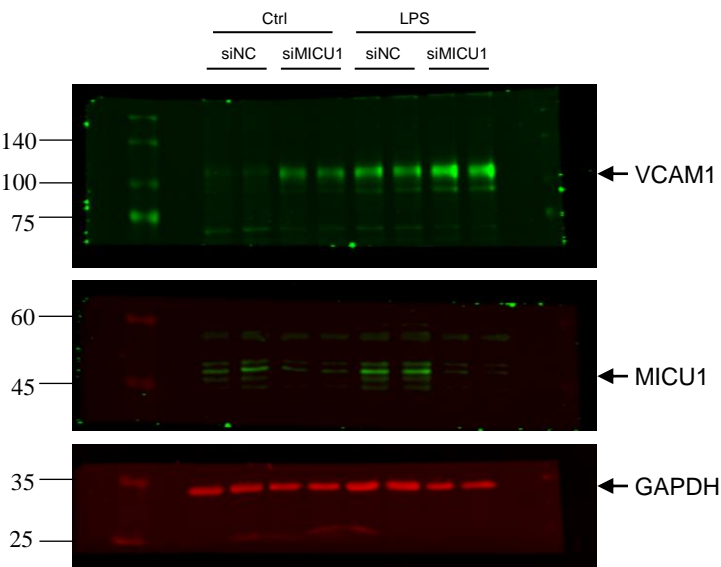

F2-L

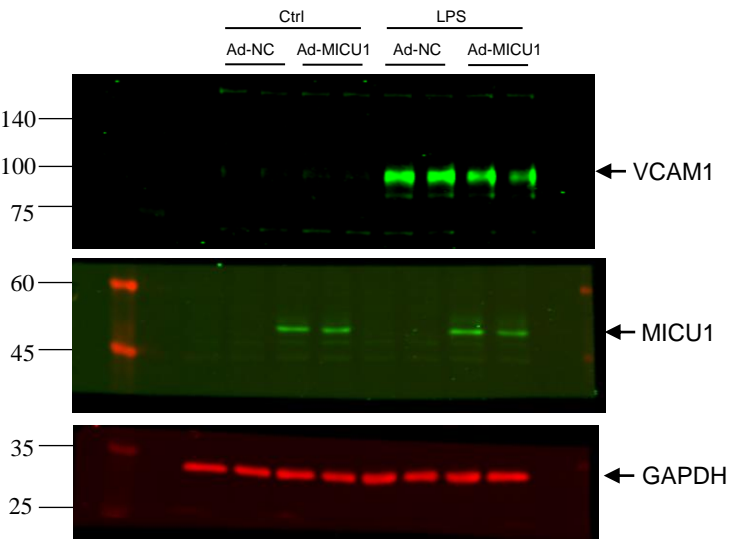

F5-A

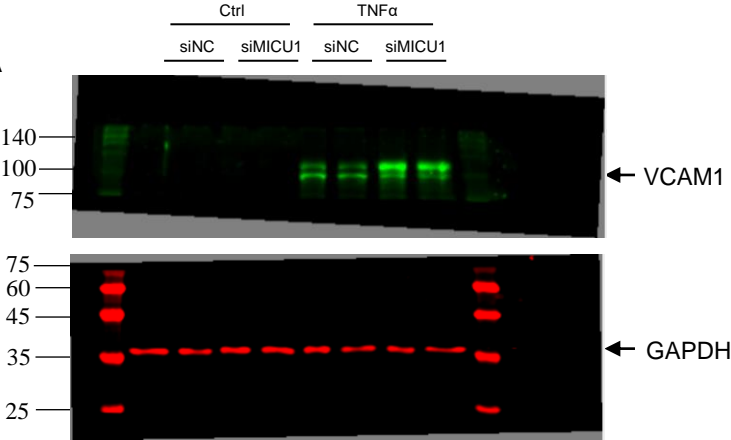

F5-B

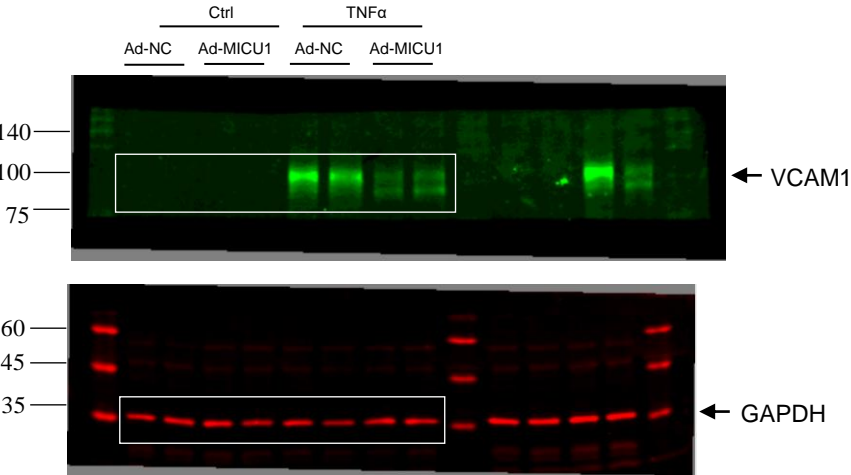

F5-C

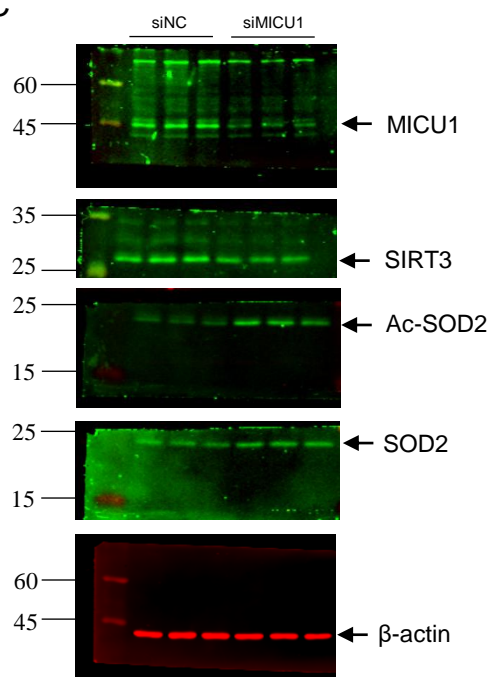

F5-D

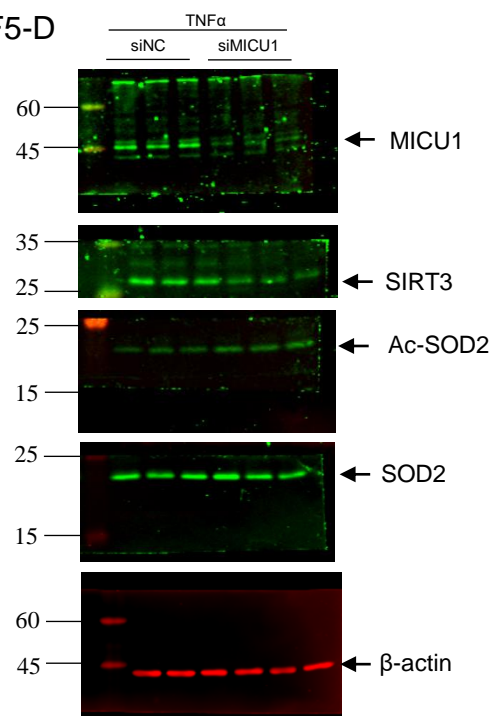

F5-E

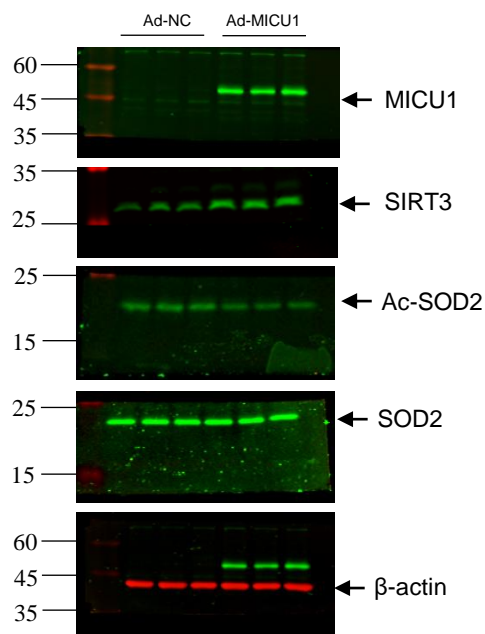

F5-F

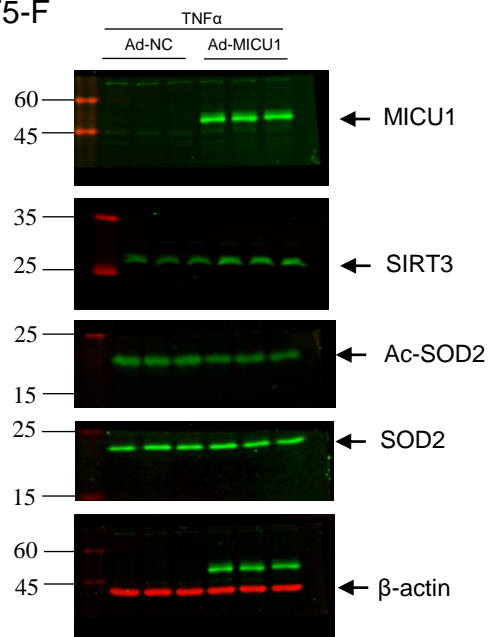

F5-G

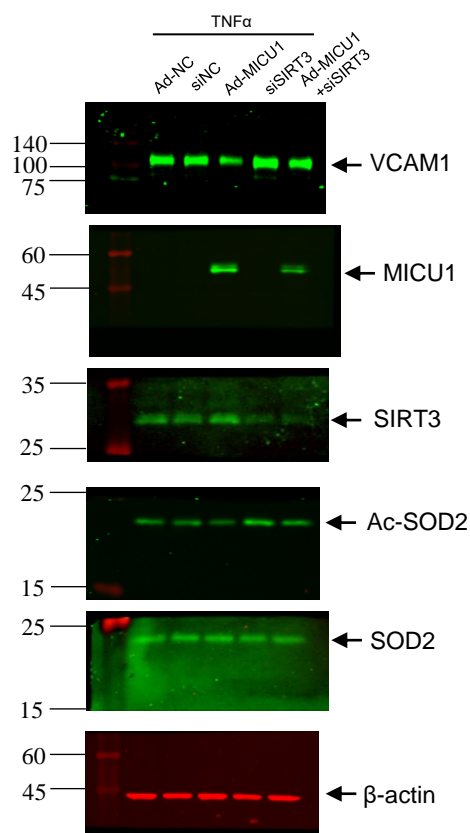

F8-I

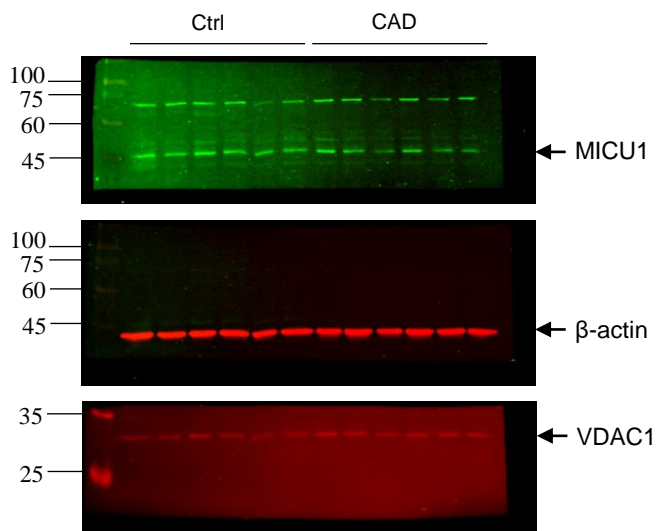

F S2-B

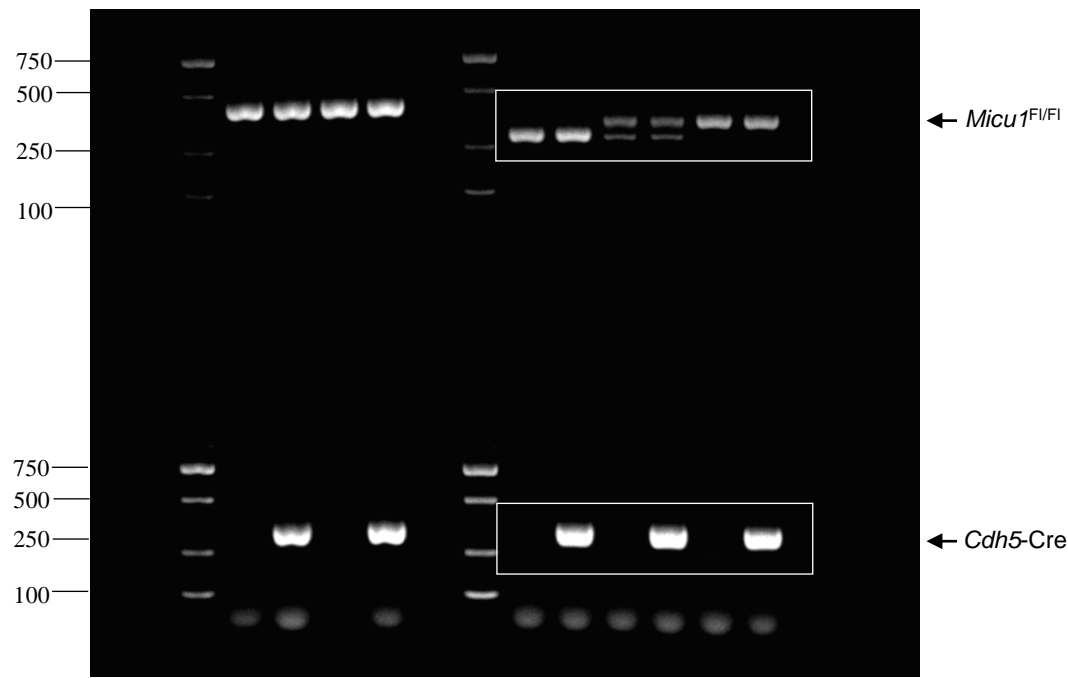

F S2-C

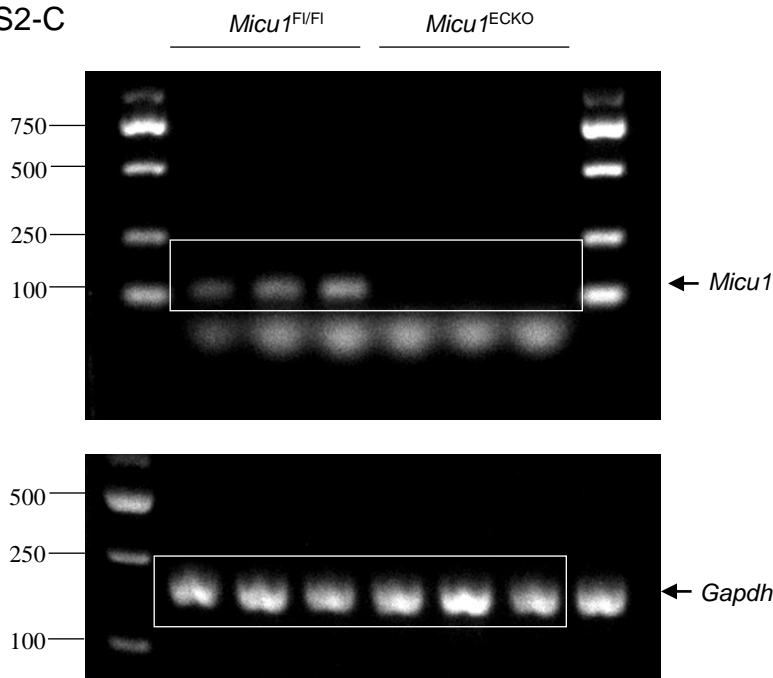

F S4-B

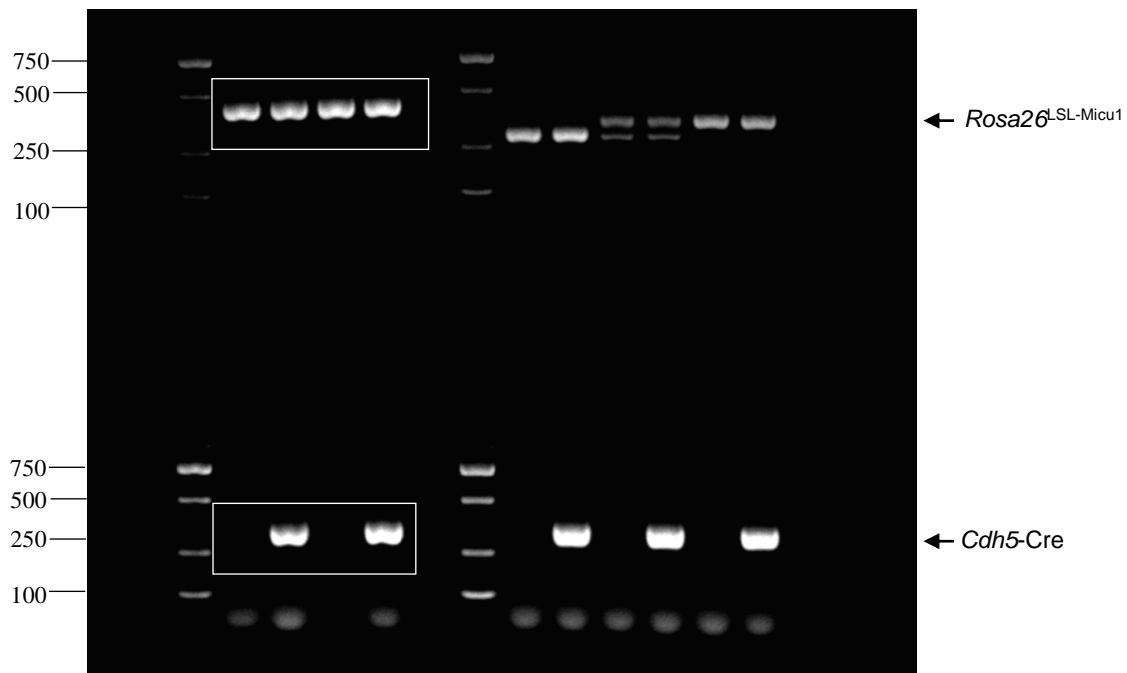

Supplement: Unedited blot and gel images [file jci-135-181928-s284.pdf]
